# Heat up and Destroy: Immunotherapy of “Cold” Tumors Using the Example of Glioblastoma

**DOI:** 10.3390/ijms27052457

**Published:** 2026-03-07

**Authors:** Yuliya Nikitina, Alina Kazakova, Maria Bogachek, Anastasia Leonteva, Natalia Vasileva, David Sergeevichev, Sergey Vladimirov, Vladimir Richter, Anna Nushtaeva

**Affiliations:** 1 Scientific Center of Genetics and Life Sciences, Sirius University of Science and Technology, 1 Olimpiysky Avenue, Krasnodar Region, 354340 Sirius, Russia; nikitina.ya@talantiuspeh.ru (Y.N.); kazakova.ala@talantiuspeh.ru (A.K.); maryambogachek@gmail.com (M.B.); anastleont@mail.ru (A.L.); vasileva.ns@talantiuspeh.ru (N.V.); sergeevichev.ds@talantiuspeh.ru (D.S.); vladimirov.sk@talantiuspeh.ru (S.V.); 2 Institute of Chemical Biology and Fundamental Medicine, Siberian Branch of the Russian Academy of Sciences, 8, Akad. Lavrentiev Avenue, 630090 Novosibirsk, Russia; richter@1bio.ru

**Keywords:** glioblastoma, tumor microenvironment, immunotherapy, CAR-T4, immune checkpoint inhibitors, NK cells, oncolytic viruses, combination therapy, systematic review

## Abstract

The characterization of tumors as either “hot” or “cold” is determined by intrinsic properties of the cancer cells, the characteristics of the tumor immune landscape, the composition of the tumor microenvironment (TME), and underlying signaling mechanisms. These biological factors are critical in defining the clinical outcomes and therapeutic responses observed in cancer patients. The TME of glioblastoma exemplifies a case of “cold” TME, which significantly hinders antitumor immunity. This constitutes the predominant rationale underlying the ineffectiveness of immunotherapy. This review provides a thorough analysis of contemporary immunotherapeutic strategies that have been developed for the purpose of altering the immunological characteristics of tumors, with a view to achieving their effective elimination. The core mechanisms of action and future clinical applications of immune checkpoint inhibitors, adoptive cellular therapy, and oncolytic viruses (OV) are delineated. A combination of preclinical and clinical evidence suggests that OV-based combinations could be an effective treatment strategy for “cold” tumors.

## 1. Introduction

According to the World Health Organization, cancer is the second leading cause of mortality worldwide [1]. In 2022, the global incidence of cancer reached 20 million new cases and 9.7 million deaths [2]. Glioblastomas (grade IV gliomas according to the WHO classification; GBM) are considered to be among the most aggressive brain tumors. In contemporary medical literature, GBM is now defined as isocitrate dehydrogenase (IDH)-wildtype diffuse adult-type astrocytoma [3], where molecular profiling complements histological assessment to confirm the diagnosis. For almost two decades since the implementation of Roger Stupp’s protocol in 2005, therapeutic interventions have yielded unsatisfactory or negligible outcomes [4]. In this regard, there is an imperative for active research and development of new effective treatments.

Immunotherapy (immune checkpoint inhibitors, adoptive cell transfer, cytokine therapy, and cancer vaccines) constitutes a treatment modality that aims to activate the immune system to combat tumors, and it has exhibited encouraging results in the fight against cancer [5,6,7]. However, the efficacy of these treatments is not uniform, with many patients and tumor types still demonstrating unresponsiveness to these interventions [8]. The efficacy of immunomodulatory strategies is contingent upon the initial immune status of the tumor and the degree of activation of innate antitumor immunity [8]. The predominant characteristic of the majority of solid tumors is that they are immunologically “cold”, a state defined by minimal infiltration of lymphocytes, particularly CD8+ T cells, and the presence of immunosuppressive cells [9,10]. This “cold” status is associated with a poor response to immunotherapy. In order to surmount this critical limitation and enhance the efficacy of immunotherapeutic approaches for patients, the primary focus of cancer immunotherapy should be on the development of strategies that can transform “cold” tumors into “hot” ones [11].

In the contemporary medical landscape, virotherapy stands as a particularly promising modality for the treatment of malignant neoplasms. Oncolytic viruses (OVs) have the capacity to selectively replicate in malignant tumor cells, actively destroying them, remodeling the immunosuppressive TME of the tumor, and activating the immune system by attracting immune cells through the action of viral and tumor antigens. Oncolytic viruses have been shown to reverse the immunological “coldness” of tumors, transforming them into “hot” states. This change renders the tumors visible and vulnerable to the patient’s own immune system [12]. This phenomenon, known as the “warming-up effect”, serves as a fundamental principle underlying the synergy of virotherapy with other antitumor immunotherapy approaches.

This review provides a comprehensive analysis of modern immunotherapeutic strategies aimed at modulating the immunological status of tumors to facilitate their elimination. The primary mechanisms of action and future applications for the use of immune checkpoint inhibitors (ICI), adoptive T- and natural killer (NK)-cell therapy, and OVs are delineated. Pre-clinical and clinical data highlight the potential for combined approaches using OVs to develop new and more effective strategies for treating “cold” tumors. This possibility is based on pre-clinical data and current clinical trials using the example of glioblastoma.

## 2. Specific Features of “Hot” and “Cold” Tumors

**Figure 1:**
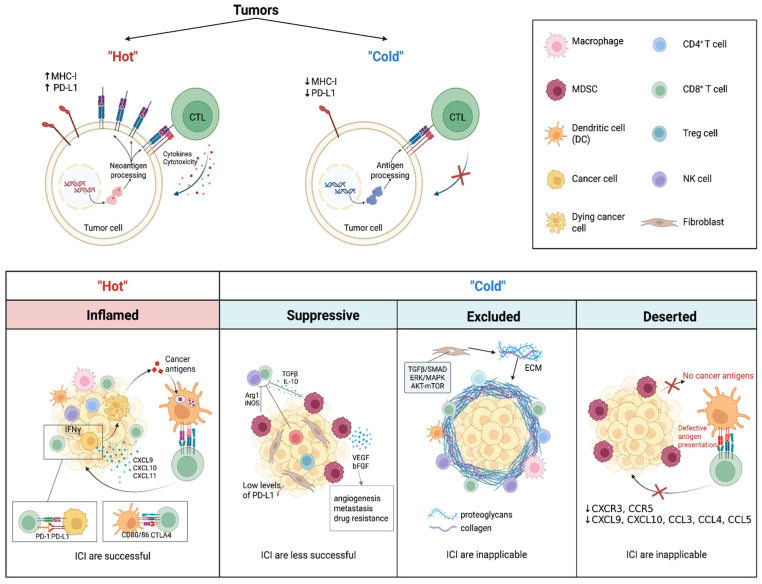
Recognized classification of tumor immunophenotypes based on the composition and spatial distribution of immune cells within the tumor microenvironment (TME). The “inflamed” (“hot”) phenotype is characterized by active infiltration of the tumor parenchyma by effector immune cells, a pro-inflammatory cytokine profile, high expression of molecules of MHC and immune checkpoint molecules, and is highly sensitive to ICI therapy. The suppressed phenotype of tumors is primarily established by suppressor cell populations, including TAM type 2, MDSC, and Treg. In a suppressive TME, the functional activity of cytotoxic T-lymphocytes is suppressed, despite their sufficient infiltration into tumors. In tumors that are excluded, T-lymphocytes are present in the tumor stroma and along its periphery; however, these cells are unable to penetrate deep into the parenchyma. The deserted phenotype is characterized by the minimal presence of immune cells in both the stroma and parenchyma of the tumor. A deficiency of tumor antigens, when concomitant with impaired antigen presentation, results in inadequate priming of T-lymphocytes and their infiltration into the tumor. The absence of activated antitumor immunity is indicative of the body’s immunological disregard of the tumor, thereby serving as a mechanism of resistance to immunotherapy. Abbreviations: ICI = immune checkpoint inhibitors, MDSC = myeloid suppressor cells, MHC = major histocompatibility complex, TAM = tumor-associated macrophage, TME = tumor microenvironment, Tregs = T-regulatory cells, ECM = extracellular matrix.

The TME is a highly dynamic and complex system in which cell composition and functional states undergo changes as the tumor progresses and in response to therapy [13]. The spatial distribution of immune cells within the TME has emerged as a critical predictor of patient outcomes and therapeutic responses [14,15]. The most common classical method for determining tumor topography is immunohistochemistry (IHC), which enables the quantitative assessment of immune cell type, density and localization in relation to other cell types [16]. The classification of the immune response within the TME into “inflamed”, “suppressive”, “excluded”, or “deserted” (Figure 1). These immunophenotypes (TIME) have been shown to correlate with response to therapy [17,18,19].

Immune-inflamed tumors, also referred to as “hot” tumors, are characterized by lymphocytic infiltration within the tumor parenchyma, with immune cells situated in close proximity to tumor cells (Figure 1). However, in this particular type of TIME, immune responses are suppressed by the activation of immune checkpoints [20,21]. Tumor cells express ligands on their surface that bind to inhibitory receptors on immune cells, thereby acting as immune checkpoints. The binding of a ligand to the receptor has been demonstrated to result in the activation of downstream signaling pathways and the negative regulation of the immune response. CD8+ T cells undergo a loss of effector functions, characterized by a disruption in their proliferative and cytotoxic activity, and a reduction in the production of cytokines that are essential for the elimination of cancer cells [22,23]. This process enables the tumor to evade immune surveillance [24,25]. However, the high expression of ligands for inhibitory receptors renders “hot” tumors more sensitive to ICI [26]. At the molecular level, the immune-inflamed phenotype is associated with an active interferon-gamma (IFN-γ) signaling signature, accompanied by increased production of chemokines, including CXCL9, CXCL10, and CXCL11. These mediators play a pivotal role in the recruitment of effector T cells into tumor tissue. Other salient features of immune-inflamed tumors encompass genomic instability, elevated tumor mutational burden (TMB), and pronounced expression of major histocompatibility complex (MHC) class I and II molecules [27,28].

Immune-desert tumors are distinguished by the absence or extremely low number of CD8+ T lymphocytes, both within the tumor and at its periphery (Figure 1). Nonetheless, the presence of myeloid-derived suppressor cells (MDSC) has been observed [29]. The phenomenon of “desertification” has been observed to occur as a result of low expression of specific chemokines and their corresponding receptors, such as CXCR3 and its chemokine ligands, namely, CXCL9 and CXCL10 [30], CCL3, CCL4, and CCL5 [31]. The absence of tumor antigens, in conjunction with defects in the antigen-processing and presentation system, has been demonstrated to inhibit T-cell priming and migration in the tumor bed. This, in turn, prevents effective immunotherapy [27].

In the context of immune-suppressive tumors (Figure 1), the TME is characterized by the predominance of immunosuppressive cell populations, including tumor-associated macrophages (TAM) phenotype 2, MDSC, T-regulatory cells (Tregs), suppressor B cells, and cancer-associated fibroblasts (CAF) [32,33]. TAM phenotype 2, by secreting cytokines IL-10 and transforming growth factor (TGF)-β, has been shown to inhibit the activation and proliferation of cytotoxic T lymphocytes (CTLs) and NK cells [34,35]. This results in an immunosuppressive TME that fosters tumor evasion of immune surveillance. The capacity of MDSCs to generate reactive oxygen species (ROS), arginase-1 (Arg-1), nitric oxide synthase (iNOS), and other immunosuppressive factors results in the suppression of T-lymphocyte activation, and the expression of MDSCs immune checkpoints leads to immune evasion of the tumor. The secretion of vascular endothelial growth factor (VEGF) and basic fibroblast growth factor (bFGF) by MDSCs, in conjunction with the activation of matrix metalloproteinases (MMPs) in these cells, has been demonstrated to contribute to angiogenesis, metastasis and resistance to therapy [36]. The synergistic effect of these factors culminates in immune effector cell depletion and diminished infiltration, consequently impeding effective elimination and tumor destruction.

Immune-excluded tumors are distinguished by an accumulation of immune cells within the TME (Figure 1). However, these cells are confined to the tumor stroma and do not penetrate the tumor parenchyma [15]. The activation of transforming growth factor-beta/Sma- and Mad-related proteins (TGF-β/Smad), extracellular signal-regulated kinase/mitogen-activated protein kinase (ERK/MAPK) and protein kinase B/mammalian target of rapamycin (AKT/mTOR) signaling pathways in the TME has been shown to promote the activation and differentiation of fibroblasts, leading to the generation of the extracellular matrix (ECM) and the formation of a dense physical barrier around the tumor [37,38,39]. Key ECM components, such as collagens, hyaluronic acid, glycoproteins and proteoglycans, influence the qualitative and quantitative composition of immune cells in the TME, contributing to tumor evasion of immune surveillance [40]. The formation of a dense network by collagen results in an increase in stromal stiffness. The subject in question exerts a significant influence on cell migration pathways and effectively prevents the infiltration of cytotoxic immune cells. Moreover, it contributes to their functional exhaustion [41]. A high hyaluronic acid content has been demonstrated to restrict the infiltration of NK cells into the tumor and to suppress antibody-dependent cell-mediated cytotoxicity (ADCC) [42]. The disruption of immune cell migration leads to the exclusive localization of CD8+ T lymphocytes to the periphery of the tumor invasion. These cells have been observed to remain within the stromal compartment, exhibiting a pattern of resistance to penetration of the parenchyma or infiltration of the primary tumor mass [43].

The enhancement of immune activation and the induction of cell death represent a therapeutic objective for tumors that are both “cold” and “hot”. However, achieving this objective necessitates the precise stratification of tumor phenotypes [44]. It is imperative to accurately differentiate between “cold” and “hot” tumors, as this is fundamental for predicting response to therapy and guiding treatment decisions. This differentiation should be based on tumor-intrinsic factors, immune landscape, and TME characteristics.

## 3. Glioblastoma as a Tumor with a “Cold” Phenotype

In the case of “cold” tumors, such as high-grade gliomas, it is imperative to adopt approaches that are aimed at eliminating the effects of immunosuppressive cells and their mediators. Conventional wisdom regarding the efficacy of immunotherapeutic approaches for the treatment of brain tumors had previously been that they were ineffective due to the prevailing theory of the immunologically privileged location of the central nervous system (CNS), protected by the blood–brain barrier (BBB), lacking specific antigen-presenting cells, and with limited access to T cells. However, this perspective has been challenged by the findings regarding the lymphatic system in the brain and the capacity of microglia/macrophage cells to present antigens, thereby activating lymphocytes following disruption of the BBB [45,46].

**Figure 2:**
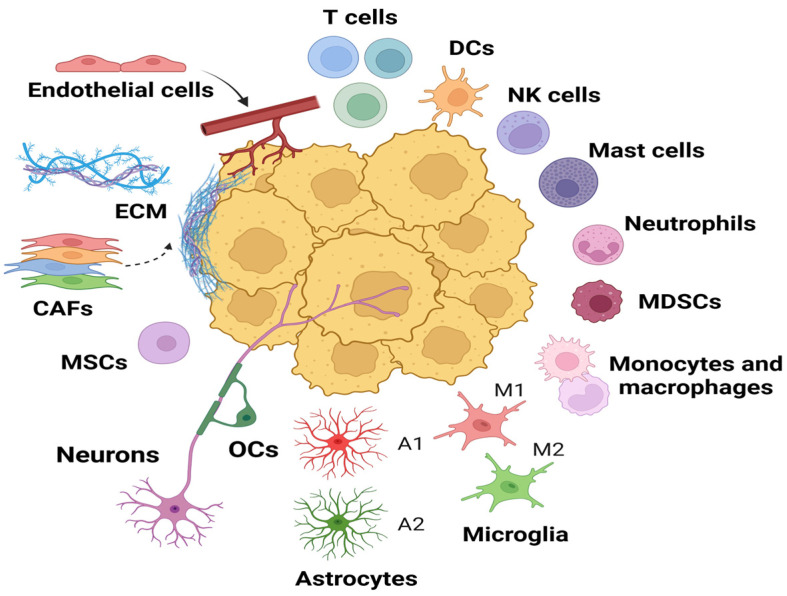
TME of glioma. The glioma TME comprises various cell types, including resident glial cells (astrocytes and oligodendrocytes), neuronal cells, endothelial cells, and CNS-resident immune cells such as microglia. Furthermore, infiltrating immune populations, including TAMs, T cells, and myeloid-derived suppressor cells (MDSCs), are abundant within the TME. In addition to immune cells, the TME encompasses the extracellular matrix, vasculature, and stromal cells, all of which play critical roles in supporting tumor growth and invasion. Abbreviations: CAFs = cancer-associated fibroblasts, DCs = dendritic cells, ECM = extracellular matrix, MDSC = myeloid suppressor cells, MSCc = mesenchymal stromal cells, NK = natural killer cells, OCs = oligodendrocyte, TME = tumor microenvironment.

The aggressiveness of glioblastoma is determined not only by cellular interactions, but also by its ability to actively undergo metabolic and chemotactic remodeling of the TME (Figure 2) [47]. Tumor cells secrete a wide range of chemokines, cytokines, and growth factors that attract immunosuppressive cells (MDSCs, TAM phenotype 2, T-helpers (Th) of the 2 types, Tregs), forming a “cold” TME [24,48,49,50]. The metabolic shift has been determined to not only promote tumor growth, but also to disrupt the function of infiltrating immune cells [51]. GBM cells have been observed to produce CCL2, CCL5, CXCL, and SDF-1, which act as attractants for TAM [52]. Under conditions of low oxygen concentration, low glucose levels, and high lactate content (TME conditions), these TAM polarize into a pro-tumor M2-like phenotype. This, in turn, has been shown to contribute to tumor progression, invasion, and migration [53]. Acidification of the ECM has been demonstrated to suppress CD8 T cell proliferation and cytotoxic function [54].

During the development of gliomas, key stromal components, including astrocytes, microglia, and cancer-associated fibroblasts (CAFs), become the tumor’s primary line of defense against immune surveillance and therapeutic effects (Figure 2). Despite their minimal presence in healthy nervous tissue, CAFs play a pivotal role in the development of immunosuppressive and therapeutically resistant TME GBM. CAFs have been shown to produce and remodel the extracellular matrix (ECM), thereby establishing a dense physical barrier that restricts the access of immune cells to the tumor [55]. The chemokine CCL2, secreted by CAFs and binding to the CCR2 receptor on GBM cells, has been shown to promote ERK1/2 expression and attenuate temozolomide-induced apoptosis, thereby increasing tumor chemoresistance [56]. Furthermore, CAFs have direct immunosuppressive effects, through the secretion of TGF-β and PGE2. CAFs can suppress the activity of cytotoxic T- and NK-cells and can also promote the expansion of regulatory-T cells [55]. The activation of the Jagged1-Notch axis induces expression of PD-L1 immune checkpoints on CAFs, which in turn leads to depletion of T-lymphocytes and the consequent evasion of tumors from immune surveillance [55]. In addition to directly suppressing immunity, CAFs contribute to GBM progression by enhancing the migration and invasive potential of malignant cells [57], as well as supporting glioma stem cells (GSCs) [58].

In a healthy brain, astrocytes perform vital homeostatic, metabolic, structural, immune, and neuroprotective functions [59,60]. However, under the influence of GBM, astrocytes undergo functional changes and acquire a reactive phenotype [61]. Hypoxia and oxidative stress in TME exacerbate this process, leading to astrogliosis and the formation of a glial scar. This scar restricts the penetration of therapeutic agents into the tumor parenchyma. In GBM, the reactive astrocyte phenotype contributes to the creation of an immunosuppressive tumor niche. A wide range of immuno-inhibitory factors have been identified, including tenascin-C, GDF-15, TGF-β and IL-10, which have been demonstrated to inhibit the migration and function of key effector cells, namely T-lymphocytes, NK cells and dendritic cells [62,63]. The suppressive effect is enhanced by activation of specific signaling pathways. In particular, hyperactivation of the JAK/STAT pathway induces PD-L1 expression on astrocytes, providing direct inhibition of T cells [64]. The activation of key signaling pathways, namely IL-6/JAK/STAT, NF-kB, Sonic Hedgehog, p53, and PI3K/Akt, which mediate interactions between astrocytes and GBM, have been shown to promote GBM survival, invasiveness, proliferation, and angiogenesis [65,66]. In the context of TME, astrocytes assume a direct role in a process of carcinogenesis, exerting influence that extends to other cellular components. The expression of CCL2 and CSF1 by astrocytes modulates the activity of microglia, which, in turn, by releasing IL-1a and TNF, maintains the reactive state of astrocytes [67,68]. From a functional standpoint, microglia associated with gliomas exhibit a notable degree of plasticity with regard to their effector functions. The pro-inflammatory phenotype M1 (with secretion of IL-1b, TNF-α, IL-6, CCL, ROS, NO) demonstrates antitumor potential [69,70]. However, the tumor systematically shifts the balance towards the alternative, immunosuppressive phenotype M2 (secreting TGF-β, IL-10, Arg-1, Ym1, and CD36) [71]. Tumor cells and astrocytes secrete a variety of cytokines, including M-CSF, IL-4, and IL-10. These cells also release extracellular vesicles that reprogram microglia into the M2 phenotype [72,73]. Conversely, M2-polarized microglia emerge as a catalyst of tumor progression, secreting stress-induced protein 1 (STI1), epidermal growth factor (EGF), TGF-β, IL-6 and MMP-2, as well as MMP-9, directly promoting the proliferation, migration and invasion of GBM cells [72]. Hyperactivation of the STAT3 signaling pathway, a frequent occurrence in GBM, has been observed to result in excessive IL-10 secretion and pronounced immunosuppression of the TME [74]. The effect is potentiated by the production of IL-10 by alternatively activated TAM phenotype, further inhibiting CTL and NK cell activity [34]. Microglia regulate local myeloid and immune cells, in addition to maintaining systemic immunosuppression, via the production of IL-1 and TGF-β [75] and promotes the recruitment of immunosuppressive cells (Tregs, Th17, MDSC) [76].

**Figure 3:**
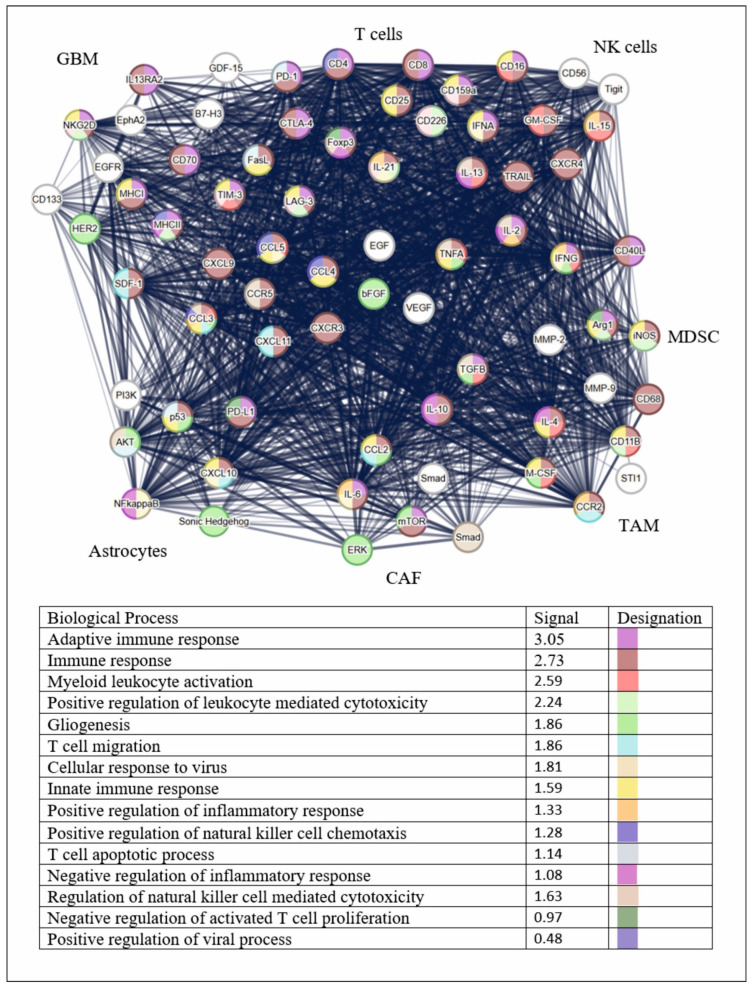
A network of protein–protein interactions in the GBM microenvironment. STRING: the proposed network of interacting glioblastoma and TME proteins [77]. The STRING network is a tool that displays protein–protein interactions in the GBM microenvironment. The nodes of the network are colored according to the participation of proteins in biological functions and are spatially arranged by cell type based on the mentions in this review. Further data can be found in Table S1. Abbreviations: GBM = glioblastomas, CAFs = cancer-associated fibroblasts, MDSC = myeloid suppressor cells, NK = natural killer cells, TAMs = tumor-associated macrophage.

The network of protein–protein interactions, determined using the STRING database (Search Tool for the Retrieval of Interacting Genes/Proteins) [77], which combines information on predicted and experimentally confirmed interactions between proteins, demonstrates a high degree of functional connectivity between expressed and secreted molecules in immunosuppressive TME GBM (Figure 3).

The network encompasses proteins of diverse functional categories, including oncogenes (EGFR, HER2), transcription factors (p53, NF-kappa B), signaling pathways (PI3K/AKT/mTOR, RAS/ERK), cytokines and chemokines (IL-6, TNFa, IFNy), immune checkpoints (PD-1/PD-L1, CTLA-4) and molecules of the major histocompatibility complex (MHC I/II) (Table S1). These data illustrate the heterogeneity of the tumor and the TME. The network density and high degree of nodes indicate that the presented set of proteins is a biologically integrated system of molecules that actively interact in multilevel signaling networks, controlling key processes in the TME (Table S2). As illustrated in Figure 3, the intricate network of intercellular interactions is depicted as a series of listed biological processes.

The interaction between intracellular proteins, cell surface receptors, and immune molecules indicates a direct effect of TME on the internal mechanisms of the tumor. This phenomenon constitutes a closed feedback loop, wherein external signals trigger internal pathways, which subsequently modulate the expression of molecules interacting with the microenvironment. The complexity and heterogeneity of the tumor structure render standard monotherapy, which targets standard monotherapy a single protein or pathway, ineffective. Tumors often develop resistance to therapy rapidly. This phenomenon can be attributed to the compensatory activation of alternative signaling pathways or alterations in the tumor’s microenvironment. Therefore, effective treatment strategies must encompass both the heterogeneity of the tumor and that of the TME. Thus, it is obvious that effective GBM therapy will be based on a combined approach, where treatment will be developed based on an understanding of the molecular and immune architecture of the patient’s tumor, and will include multicomponent, synergistic combinations of drugs acting on different levels of this complex network.

## 4. The Strategy of “Cold”–“Hot” Tumor Transforming

Recent advances in immunotherapy, including checkpoint inhibitors, CAR-T or NK- cells, and oncolytic viruses, have demonstrated the capacity to effectively counteract immunosuppression in the TME, thereby significantly enhancing the antitumor response. The following discussion will address each strategy in turn.

### 4.1. Checkpoints Inhibitors

Immune checkpoints (ICI) are defined as inhibitory receptors located on immune cells that function by signaling the activation of immunosuppressive pathways. These checkpoints are imperative for the maintenance of self-tolerance and the modulation of immune responses, their severity, and duration. The category of inhibitory receptors of signaling pathways includes PD-1, CTLA-4, T-cell immunoglobulin and TIM-3, and LAG-3. The extant experimental data indicate that the interaction of PD-L1 on tumor cells with the PD-1 receptor on T lymphocytes results in a significant suppression of their activation and a substantial decrease in the production of key cytokines, including IFN-γ, IL-2, and IL-10 [78]. Furthermore, glioma cells have been observed to enhance PD-L1 expression in circulating monocytes and TAMs [47]. PD-L1 expression is present on microglial cells in human GBM samples [79]. Consequently, the inhibition of the PD1–PD-L1 axis has significant therapeutic potential in tumor therapy [80]. The integration of ICI in GBM has been found to enhance immune system function, thereby overcoming immune tolerance and stimulating the primary components of the immune response, including effector CD8+ T lymphocytes, helper CD4+ cells (Th1), and NK cells, which collectively contribute to the effective destruction of the tumor [81]. The combination of ICI with conventional GBM treatments, radiation, and chemotherapy has also been employed to enhance treatment efficacy [82]. However, phase III studies of the combination of nivolumab (anti-PD-1) with radiation therapy did not demonstrate any advantages of ICI over standard temozolomide (TMZ) therapy with radiotherapy [83]. In addition, the results of a study on patients with methylated O6-Methylguanine-Methyltransferase status revealed that the addition of nivolumab into the TMZ+radiotherapy regimen did not provide a significant enhancement in outcomes, even when compared to placebo [84].

Nevertheless, despite the negative results, a number of studies have indicated the presence of persistent clinical responses to immunotherapy [85]. Tanner M. Johanns` research revealed that the treatment of patients diagnosed with GBM resulted in the activation of the immune system within the CNS, accompanied by clinically and immunologically significant responses [86]. Neoadjuvant therapy with the anti-PD-1 agent, pembrolizumab, prior to planned surgical resection, followed by an adjuvant course of the same drug, has been shown to result in a significant increase in overall survival (OS) and recurrence-free survival (RFS) in patients with recurrent GBM [87]. In addition to studies employing PD-1 antibodies, the efficacy and safety of PD-L1 antibodies (e.g., durvalumab, atezolizumab, and avelumab) have also been evaluated in patients with GBM. The results demonstrated the potential for antitumor efficacy and good tolerability [88,89,90]. The combination of atezolizumab, radiation therapy, and TMZ has shown a high degree of efficacy in prolonging survival in patients diagnosed with newly diagnosed GBM. The efficacy of the therapy was contingent upon the baseline level of CD4+ T cells [91].

Recent studies in the domain of ICI immunotherapy have witnessed an escalating focus on novel and promising targets, including LAG-3, TIM-3, TIGIT, and IDO1. Two phase I clinical trials (NCT02658981 and NCT03493932) are currently underway to evaluate the activity of the LAG-3 inhibitor relatlimab (BMS-986016) in GBM therapy. The primary objective of these trials is to ascertain the activity of the drug as monotherapy and in combination with nivolumab. In preclinical studies, dual blockade of LAG-3 and PD-1 has been proven to enhance T-cell activation and promote a more potent antitumor immune response [92]. TIM-3 is another immune checkpoint receptor that plays a key role in T cell exhaustion. The over-expression of TIM-3 in T cells and myeloid cells in GBM has been associated with immune evasion [93]. In the course of preclinical studies conducted by Kim, Jennifer E., the combination of anti-TIM-3 and anti-PD-1 therapies demonstrated a synergistic effect on the inhibition of tumor growth and the activation of T cells specific to tumor antigens. This approach led to long-term survival in mouse glioma models [94]. A study of the effects of dual blockade of TIM-3 (sabatolimab) and PD-1 (sparalizumab) in combination with stereotactic radiosurgery is currently in Phase I clinical trials. (NCT03961971). The effects of TIGIT inhibitors in GBM therapy are also being actively investigated (NCT04656535, NCT04047706, and NCT02052648). TIGIT has been identified as a critical regulator of tumor recognition by T cells and NK cells. The efficacy of dual PD-1/TIGIT blockade in enhancing the number and functional activity of tumor antigen-specific CD8+ T cells in vitro has been established, concurrently promoting tumor elimination in murine models [95]. A number of studies are underway to assess the safety and efficacy of anti-PD-1/TIGIT combination therapy in Phase I clinical trials (NCT04047706, NCT04656535, NCT02052648).

The extant data demonstrate the efficacy of these strategies in enhancing patient outcomes. However, despite the advancements in immunotherapy and the ongoing clinical trials, there is currently no FDA-approved ICI therapy for GBM.

### 4.2. Cellular Immunotherapy

Adoptive immunotherapy is recognized as one of the most promising methods for treating malignant neoplasms, due to its high effectiveness and promising research results. The following categories are recognized as the primary forms of adoptive cell therapy: T-cell therapy, CAR-T- or NK-cell therapy, and immunotherapy based on macrophages and dendritic cells (DCs).

### 4.3. Tumor-Infiltrating Lymphocytes

The focus of immunotherapy is frequently on the use of tumor-infiltrating lymphocytes (TILs). The process entails the isolation of lymphocytes from a resected tumor, their ex vivo expansion with high-dose recombinant IL-2, and, when necessary, their priming with cytokines such as IL-15 and IL-21. These expanded cells are subsequently reintroduced into the patient [96]. A preliminary investigation into the safety of autologous TILs therapy in combination with IL-2 for recurrent malignant gliomas revealed limited antitumor activity, likely attributable to TILs heterogeneity and TCR variability [97]. The aforementioned study will assess the safety and efficacy of TILs therapy for the treatment of gliomas. This objective will be achieved by conducting Phase I clinical trials (NCT05333588 and NCT04943913) that are currently underway. In addition, autologous TILs that secrete antibodies targeting PD-1 have been developed. Patients with GBM exhibited a positive response to PD-1-TILs therapy, exhibiting high antitumor efficacy in comparison to conventional TILs [98]. “Cold” tumors, defined by inadequate T-cell infiltration and diminished MHC molecule expression session, frequently exhibit resistance to TILs therapy.

### 4.4. CAR-T Therapy

The basis of CAR-T therapy is the generation of tumor-specific cytotoxic lymphocytes that express a CAR. In recent years, there has been a marked increase in research activity concerning the use of CAR-T therapy for the treatment of GBM. A review of the literature reveals that the most promising molecular targets utilized in CAR-T therapy for gliomas include epidermal growth factor receptor variant III (EGFRvIII) [99,100], IL-13Rα2 [101,102,103] and HER2 (NCT03500991) [104,105], erythropoietin-producing hepatocellular A2 (EphA2) [106]. Other notable targets include CD276 (B7-H3, a member of the B7 family of immunoregulatory proteins), CD70, chlorotoxin, IL-7Rα, disialoganglioside 2 (GD2), MMP-2, and integral membrane protein type II (NKG2D) [107].

#### 4.4.1. Anti-IL13Rα2-CAR-T Therapy

The IL-13 receptor α2 (IL-13Rα2) is expressed in a wide variety of tumor cells from patients diagnosed with GBM, and it functions as a prognostic indicator of poor patient survival. The potential of IL-13Rα2 as a CAR-T target is further strengthened by its lack of expression in normal brain tissue [108].

In 2015, an initial safety and feasibility study was conducted on the treating of recurrent GBM with CAR-engineered T cells targeting IL13Rα2. The treatment was well tolerated and resulted in transient antitumor activity [101]. The patient’s clinical benefit was sustained for a period of 7.5 months following the initiation of CAR-T cell therapy (NCT02208362). However, the disease continued to progress due to the emergence of new foci [103,109]. It was demonstrated that IL13Rα2–CAR-T cells, through IFN-γ signaling, activate endogenous T cells, monocytes/macrophages, and trigger tumor-specific T-cell responses in patients diagnosed with GBM [110].

#### 4.4.2. Anti-EGFRvIII-CAR-T Therapy

EGFRvIII is a mutant variant of the epidermal growth factor receptor (EGFR) that is expressed on the surface of tumor cells in 30–40% of GBM cases [93,108,111,112,113,114]. A deletion in the EGFR gene results in ligand-independent constitutive activity of the EGFR pathway [113]. Moreover, the mutant form of the receptor, EGFRvIII, has been demonstrated to shape an immunosuppressive TME, and promote tumor evasion of T-cell and NK cell immune responses. The pathogenic effects of EGFR have rendered it a promising target for therapy [115]. In studies employing orthotopic TMZ-resistant mouse models of GBM, combination therapy involving dose-intensified regimen TMZ and CAR-T cells resulted in substantial tumor regression [116]. In a group of patients with recurrent GBM, atezolizumab was characterized by its good tolerability. Furthermore, the efficacy of the therapy was contingent upon the number of circulating CD4+ T cells (NCT02664363). A phase I study, designated as NCT02209376, employed CAR-T-EGFRvIII, thereby providing evidence regarding the safety and target activity of CAR-T cells in the brain. However, in situ assessment of the TME revealed a compensatory development of immunosuppressive response by T cells expressing CD4, CD25, and Foxp3, as well as increased expression of inhibitory molecules, in particular IDO1, PD-L1, and IL-10 [100]. The combination of anti-EGFRvIII CAR-T-cell therapy with pembrolizumab in patients with recurrent GBM did not exhibit a clinically significant response (NCT03726515).

The hypothesis that tumor cells evade CAR T-cell-based EGFRvIII targeting approaches is predicated on the premise of high expression heterogeneity on tumor cells [117]. To address this issue, the CARv3-TEAM-E construct was developed, targeting the EGFRvIII receptor and wild-type EGFR via secretion of an antibody molecule that interacts with T cells. In a clinical trial (NCT05660369), intraventricular administration of CARv3-TEAM-E resulted in transient tumor regression [118].

The targeting of distinct antigens represents a complex approach for the application of CAR-T cell therapy. In a recent phase I trial (NCT05168423), intrathecal injection of bivalent CAR-T cells targeting both EGFR and IL13Rα2 demonstrated encouraging early response rates in patients with multifocal, refractory GBM. However, this trial did not meet the neuro-oncology objective response criteria [119].

#### 4.4.3. Anti-HER2-CAR-T Therapy

CAR-T cells, which are capable of recognizing HER2—a transmembrane protein and member of the EGFR family—have also been developed. The frequency of HER2 expression in GB cells is approximately 80%, positioning HER2 as a promising target for the therapy [120].

Phase 1 trials (NCT01109095) confirmed the safety of HER2-specific CAR-modified virus-specific T cells. The median OS of patients was 11.1 months after the initial T-cell infusion and 24.5 months from diagnosis [105]. Another clinical trial (NCT03500991) proved tolerability of multiple infusions of HER2 CAR-T cells and the local activation of the immune response in the CNS (NCT02442297, NCT03389230) [104]. In preclinical studies, encouraging results were observed with the use of tandem CAR-T cells targeting both HER2 and IL13Rα2 [121] and triple CAR-T cells targeting HER2, IL13Rα2, and EphA2. These CAR-T cells overcame tumor resistance due to antigenic heterogeneity and improved treatment efficacy [122].

#### 4.4.4. Anti-B7-H3 CAR-T Therapy

As an immune checkpoint protein, B7-H3 has been shown to exert both costimulatory and co-inhibitory effects on T-cell immune activation, thereby creating an immunosuppressive TME [123,124]. In preclinical studies, B7-H3-specific CAR-T cells have demonstrated encouraging outcomes [125]. The compounds exhibited a cytotoxic effect on GBM cells in vitro and significantly accelerated tumor regression in orthotropic GBM models [126].

The Phase I BrainChild-03 trial, which involved repeated intraventricular administration of B7-H3 CAR-T cells, documented good tolerability of the cell product, immune activation in the CNS, and potential clinical benefit in the treatment of diffuse intrinsic pontine glioma (DIPG) (NCT04185038) [127].

#### 4.4.5. Anti-EphA2-CAR-T Therapy

EphA2, a member of the tyrosine kinase receptor family, has been shown to play a significant role in tumor oncogenesis and the formation of a pro-tumor TME [128]. EphA2 expression is elevated in 90% of GBM cases, while it is relatively low in normal brain tissue [129]. A clinical trial (NCT 03423992) demonstrated the ability of EphA2-directed CAR-T cells to traverse the BBB and proliferate within the recurrent GBM TME. However, the clinical effect of the therapy was transient, which is consistent with the phenomenon of CAR-T-cell persistence [130].

In a study by Bielamowicz et al., second-generation multi-targeted CAR-T cells with a CD28 costimulatory domain were developed that target three antigens: HER2, IL-13Rα2, and EphA2. These CAR-T cells exhibited heightened toxicity and cytokine release in comparison to monospecific and bispecific CAR-T cells in patients diagnosed with gliomas that were positive for one of the antigens under investigation. Multi-target therapy has been proven to overcome antigenic heterogeneity and enhance effector T cell function [122].

#### 4.4.6. Anti-GD2 CAR-T Therapy

GD2 is an attractive therapeutic target due to its minimal expression in normal cells, with the exception of limited expression in brain cells and peripheral nerves, and ability to maintain GBM immunosuppression by modulating the activity of macrophages and suppressing the function of NK cells [131,132,133]. In addition, GD2 has been shown to diminish the functional activity of T cells and DCs, thereby promoting the recruitment and accumulation of MDSCs [134] and Tregs cells within the TME [135]. Inhibition of GD2 expression in glioma models has been evidenced to affect the nature and distribution of macrophages in the TME [135,136].

Preclinical studies have demonstrated the efficacy of GD2-CAR-T cell therapy in orthotopic xenograft models of diffuse midline gliomas (DMGs) with the histone H3 K27M mutation (H3-K27M). Systemic administration led to the disappearance of infiltrated tumors. However, peritumoral neuro-inflammation during the acute phase of antitumor activity resulted in hydrocephalus, which proved fatal [137]. In a recent clinical trial (NCT03170141), infusions of GD2-specific 4SCAR-T cells were well tolerated and caused partial response in patients with GD2-positive advanced GBM.

#### 4.4.7. Anti-CD70 CAR-T Therapy

CD70, a member of the tumor necrosis factor (TNF) family, is an immunosuppressive ligand overexpressed in patients with primary glial tumors and recurrent GBM [138]. In normal tissues, its expression is restricted to antigen-activated T, B, and NK cells, as well as on a small subset of mature DCs [139,140]. CD70-specific CAR-T cells exhibited high efficacy in patient-derived xenografts and syngeneic mouse tumor models [138]. CD70 CAR-T cells modified to express the IL-8 receptors, CXCR1, and CXCR2, demonstrated enhanced migration and persistence, resulting in tumor regression, inhibition of immunosuppression, and a stable antitumor response in preclinical models of GBM [141]. A Phase I clinical trial (NCT05353530) of IL-8 receptor-modified CD70 CAR-T-cell therapy is currently underway in patients with CD70-positive, MGMT-unmethylated GBM.

#### 4.4.8. NKG2D

NKG2D has been identified as an activating immune receptor [142,143,144]. In studies by Dong Yang, NKG2D-BBz CAR-T cells manifested high efficacy against GBM cells, leading to substantial elimination of xenografted tumors in vivo without significant treatment-related toxicity [145]. Nevertheless, the encouraging data on the efficacy of CAR-T therapy suggests that GBM often evades NKG2D-mediated immune surveillance by downregulating or shedding soluble ligands from the cell surface [146].

The administration of chemotherapy and radiation therapy has been demonstrated to result in an augmentation of the expression of NKG2D ligands in GBM cells. Consequently, the combination of conventional therapy with NKG2D-targeted immunotherapy is anticipated to overcome the immunosuppressive TME, sensitize antigen-negative subclones to CAR recognition, and achieve a high antitumor response [147]. In addition, a number of pharmaceuticals, including sodium valproate, gemcitabine, superpolyamide, hydroxyamino acid, and TMZ, enhance NK cell-mediated toxicity by increasing the expression of NKG2D ligands [148].

Preclinical investigations have shown that sodium valproate in conjunction with NKG2D CAR-T cells therapy has the potential to enhance antitumor activity in a GBM xenograft model in vivo [149]. The results of studies on NKG2D-based CAR-T cells in combination with radiation therapy in orthotopic syngeneic glioma models indicated a synergistic effect, characterized by the stimulation of CAR-T cell migration to the tumor site and enhancement of effector functions. These findings also demonstrated a significant increase in OS in experimental animals [150]. Hanna Meister’s research showed the pronounced antitumor activity of NKG2D CAR-T cells co-expressing IL12 and IFN-α2 in orthotopic immunocompetent glioma mouse models. The expression of IL12 and IFN-α2 promoted the formation of a pro-inflammatory TME and reduced T-cell immune exhaustion [151]. Preclinical studies are also investigating targets such as carbonic anhydrase IX (CAIX), CD70, chondroitin sulfate proteoglycan 4, fibroblast growth factor-inducible 14 (Fn14), and trophoblast cell surface antigen 2 [152].

#### 4.4.9. The Limitations and Challenges of CAR-T Therapy

CAR-T therapy is characterized by its high degree of specificity and effectiveness, suggesting a broad potential for the treatment of various types of cancer. However, despite the encouraging results, the rate of achieving clinically significant responses in GBM remains limited, and relapses are common. This phenomenon can be attributed to the unique biology of the tumor, including its antigenic heterogeneity, which enables immune evasion, an immunosuppressive TME populated by MDSCs and Tregs, and the limited infiltration and persistence of CAR-T cells within the TME [153]. A significant constraint of CAR-T therapy is the potential for the emergence of severe adverse effects, including cytokine release syndrome (CRS), hematological disorders, and neurotoxicity, which can manifest promptly and necessitate prompt medical intervention. The manifestation of CRS is primarily associated with the release of IL-6 by immune cells. The costly and labor-intensive process of producing CAR-T cells individually for each patient, as well as the inability to use allogeneic products, also limit the use of CAR-T cells in clinical trials [154].

As an alternative to CAR-T cells, a promising approach to adoptive cell therapy is the use of a subpopulation of innate immune cells, namely NK cells. These cells constitute 5–20% of all circulating human lymphocytes [155]. These cells play an important role in shaping the early immune response, possessing cytotoxic activity and mediating antiviral and antitumor reactions [156].

### 4.5. NK Cell Therapy

The efficacy, safety, and practical applicability of NK therapy are contingent upon the source of NK cells, the production method, and the modification technologies employed.

#### 4.5.1. Autologous NK Cell Therapy

In the initial clinical trials, patients diagnosed with recurrent GBM were administered infusions of autologous NK cells that had been expanded ex vivo from peripheral blood. The therapeutic agent (KCT0003815) exhibited a satisfactory safety profile and antitumor efficacy in a subset of patients; however, its clinical efficacy remained constrained. The use of ILs, particularly IL-2, IL-12, IL-15, IL-18, and IL-21, in combination with feeder cells, has been shown to augment the cytotoxic and proliferative capabilities of autologous NK cells in the context of treating malignant diseases (NCT02118415, NCT03539406, NCT03539406) [157,158]. The systemic administration of cytokines to maintain NK cell activity in vivo had serious toxic effects [159,160]. High doses of IL-2 have been observed to induce the expansion of regulatory T cells and subsequent depletion of NK cells [161]. The intracavitary administration of lymphokine-activated killer cells (LAK) and IL-2, without subsequent systemic administration, has been demonstrated to minimize the incidence and severity of adverse events. This approach enhanced long-term survival in patients diagnosed with recurrent GBM, accompanied by increased lymphocytic infiltration at the injection site [162,163]. The safety and clinical response of autologous NK cells have been observed in cases of renal cell carcinoma and breast cancer [164,165]. However, their antitumor activity was limited due to suppression by tumor cell MHC class I molecules [166,167,168].

#### 4.5.2. Allogeneic NK Cells

Allogeneic KIR-mismatched NK cells, which are capable of bypassing HLA-KIR inhibitory signals have emerged as a promising alternative to autologous cell products [169,170]. Preclinical studies have demonstrated that GSCs, which play a pivotal role in treatment resistance, exhibit heightened sensitivity to the cytolytic action of allogeneic NK cells [171,172]. The employment of donor KIR2DS2+ NK cell subsets with dominant activation signals has resulted in the effective elimination of GBM cells and the enhancement of survival in experimental models. KIR2DS2+ NK cells have been found to manifest characteristics such as intense cytokine production, a high degree of degranulation, and enhanced persistence in living organisms [170]. Furthermore, NKG2A+ NK cells have been evidenced to possess therapeutic potential within the framework of GBM immunotherapy [173].

Gene modification technologies have led to substantial advancements in the functional properties of NK cells, enhancing their resistance to the effects of an immunosuppressive TME through various mechanisms, including the stimulation of activity or the blockade of inhibitory signals [115]. The targeting of molecules that inhibit NK cell receptors (TIM-3, NKG2A, PD-1 and TIGIT) has shown potential as a therapeutic approach for GBM [174].

TGF-β, which is secreted by GSCs, activates TGF-β2 on NK cells, thereby blocking their antitumor activity [175]. TGF-β can also been observed to downregulate the expression of the key activating receptor NKG2D on peripheral blood NK cells, thereby suppressing their function [176]. The knockout of the TGF-β receptor gene (TGFBR2) in allogeneic NK cells impeded the inhibition of their function. NK cells effectively recognized and destroyed tumor stem-like cells [175]. The creation a dominant-negative TGF-β receptor II (DNTβRII) and hybrid TGF-βRII-NKG2D receptors that convert the suppressive TGF-β signal into the activating NKG2D signal has enabled the inhibition of TGF-β-mediated immunosuppressive mechanisms in mice and a significant increase in NK cell cytotoxicity (NCT04991870).

Despite the success of preclinical studies, the development of a standardized, “off-the-shelf” product based on allogeneic sources remains an unresolved challenge due to the initially low yield of NK cells and the high variability in quality between different donors [177]. The limitations imposed by technology can be categorized as follows: firstly, there is an absence of standardized protocols for ex vivo expansion and activation; secondly, difficulties are encountered in scaling; and thirdly, there is a decrease in cell functional activity after cryopreservation [178].

#### 4.5.3. iPSC-NK Cells

iPSCs are an easily adaptable platform for standardized and scalable NK cell production [179]. In addition, iPSC-NK cells demonstrate heightened in vitro cytotoxic activity against cancer cells in comparison to primary NK cells (NCT04847466, NCT04551885, NCT03841110, NCT05069935) [177]. A study by Yue Qin et al. found that NKG2A-deleted PSC-NK cells were more effective against GBM cells expressing HLA-E, a key ligand that inhibits NK cell function. The in vivo potency of the antitumor efficacy of the compound was found to significantly suppress tumor progression and extend survival in mice in xenograft models of GBM [180]. The employment of genetically modified iPSC-NK cells containing the synNotch construct, which targets the NK cell suppressor axis TIGIT/CD155 and CD73, has been demonstrated to promote the activation of natural iPSC-NK cell cytotoxicity and a pronounced antitumor response against GBM [177,181]. Notwithstanding the encouraging potential of this approach, the clinical implementation of iPSC-NK cell-based therapy is confronted with technological challenges. The prolonged production cycle (3–5 weeks) for obtaining NK cells from hESC/iPSC significantly delays the initiation of therapy, which is unacceptable for aggressive malignancies. The oncogenic potential of iPSC-NK cells remains a fundamental safety issue. Furthermore, iPSC-derived cells have the potential to elicit an immune response, which raises concerns about their use as a readily available allogeneic product [182].

#### 4.5.4. NK-Cell Lines

Standardized NK cell lines (e.g., NK-92, NK-92MI, NKL, NKG, KHYG-1, and YT) have been shown to overcome the key limitations of allogeneic NK therapy [183]. In a study by Zhang et al., modified NK-92 cells expressing HER2-specific CAR and CD28 signaling domains that activate NK cells (anti-HER2-CAR-CD28ζ) were active against HER2-positive GBM cells, including primary cultures. In immunocompetent mice, local therapy with NK-92/5.28.z cells resulted in the elimination of transplanted syngeneic tumors, the induction of endogenous antitumor immunity, and long-term protection against relapse [184]. CAR2BRAIN Phase I clinical trial (NCT03383978) showed a favorable safety profile with CAR-NK cells (NK-92/5.28.z) in patients with recurrent HER2-positive GBM. The degree of CD8+ T-cell infiltration of recurrent tumor tissue was found to be positively correlated with the duration of the progression-free period (7 weeks) [185]. The combination of HER2-targeted CAR-NK cells, NK-92/5.28.z, with anti-PD-1 checkpoint inhibitors demonstrated a significant synergistic effect, leading to tumor regression and long-term survival in vivo [186,187]. Modified NK-92 cells expressing a CAR-T targeting EGFR or its mutant form, EGFRvIII, demonstrated high specific toxicity against GBM models in both in vitro (GL261, SKMG-3 and BS153 cell models) and in vivo (LNT-229/EGFRvIII model) [188].

The employment of dual targeting of EGFR/EGFRvIII using CAR-NK cells has emerged as a promising strategy for adoptive immunotherapy of GBM [189]. Murakami et al. utilized the KHYG-1 cell line, which was isolated from the peripheral blood of a patient diagnosed with aggressive NK cell leukemia. The authors generated transduced KHYG-1 cells using a lentiviral construct encoding a CAR-T against EGFRvIII, along with the costimulatory domains CD28 and 4-1BB. The presence of CD28 provided a potent signal that activated NK cells, enhanced the production of cytokines and cytotoxic granules, and stimulated NK cell proliferation in vivo. The resulting CAR-KHYG-1 cells exhibited high specificity and toxicity against EGFRvIII-positive U87 cells, thus suggesting them as a promising therapeutic option for GBM [190]. Müller and colleagues pursued an alternative strategy for enhancing the efficacy of NK therapy via human YTS NK cells [191]. They successfully generated NK cells that exhibited enhanced CXCR4 EGFRvIII-specific CAR expression. In models of GBM secreting the XCL12/SDF-1α ligand, it was found that the resulting effector cells had increased cytotoxic activity against GB cells. Furthermore, expression of the CXCR4 receptor stimulated the recruitment of NK cells to the tumor site, thereby enhancing the antitumor response [192]. A research team led by D.V. Yuzhakova created YT-based cell lines overexpressing a modified VAV1 protein, a regulator of NK cell activity. Inactivation of the *CISH* gene in VAV1 blocked the development of NK cell resistance to IL-15 [193], while knockout of *B2M* disrupted the structure of MHC class I on the NK cell surface, rendering them “invisible” to recipient alloreactive T cells, thereby minimizing the risk of rejection and premature NK cell elimination. In vitro studies revealed that YT-VAV1+ lines exhibited enhanced cytotoxic properties. YT-CISH –/– demonstrated a substantial augmentation in efficacy in eradicating primary GBM cells in comparison to the wild-type YT [194,195]. The advent of scientific advances in preclinical studies and the employment of innovative methodologies for the modification of NK cells, encompassing the enhancement of persistence, toxicity, recruitment, and resistance to inhibitory effects, has rendered NK cell therapy as one of the most promising immunotherapy approaches for GBM [196].

#### 4.5.5. Advantages of NK Cell Therapy

NK cells, when utilized as effector cells in immunotherapy, present several advantages and have the capacity to overcome the key limitations of CAR-T cells [142]. The enhanced safety profile of NK cell therapy is marked by a reduction in neurotoxicity and CRS, attributable to the absence of IL-6 release, along with the limited lifespan of NK cells [197,198,199,200].

The safety of NK therapy is further substantiated by clinical trial data, which demonstrates its non-incidence of graft-versus-host disease and minimal risks of alloreactivity [201]. This phenomenon can be attributed to the fact that NK cell activation is not contingent upon TCR-mediated recognition of foreign HLA-peptide complexes. The transmission of inhibitory signals through NK cell receptors, killer-cell immunoglobulin-like receptors (KIR) and NKG2A, provides an additional layer of protection for healthy tissue. In contrast, KIR mismatch has been shown to enhance the antitumor activity of NK cells. These properties enable the creation of standardized, allogeneic, ready-to-use (off-the-shelf) NK cell-based products that demonstrate effectiveness even under HLA-incompatible conditions [202].

A notable benefit of NK cell therapy is its capacity to target tumors and their TME through multiple mechanisms. Firstly, NK cells have the capacity to recognize stress-induced ligands present on the surface of tumor cells, thereby exerting a direct cytotoxic effect [203]. Upon activation, NK cells engage in synapse formation with target cells, releasing cytoplasmic granules containing perforin and granzyme B. This process leads to the direct lysis of target cells [204]. Reduced MHC class I expression, a hallmark of solid tumors, results in diminished activation and function of CTLs while maintaining unaltered NK cell toxicity [205]. Furthermore, studies have demonstrated that NK cells can mediate antibody-dependent cell-mediated cytotoxicity (ADCC) through Fc receptors (FcγRIIIA/CD16 and FcγRIIC). These receptors possess the capacity to discern the Fc fragment of antibodies (predominantly IgG1) that have become bound to antigens on the surface of target cells. Activated NK cells release cytotoxic factors and lyse target cells without requiring prior sensitization. The secretion of IFN-γ by NK cells stimulates the recruitment and activation of adaptive immune cells [206]. Studies have demonstrated that NK cells are capable of executing a cytotoxic function through the expression of pro-apoptotic death ligands, such as FasL (CD95L) and TRAIL. The interaction of these molecules with receptors on the target cell membrane initiates a signaling cascade that results in cell apoptosis [207]. In addition to direct cytolytic action, NK cells secrete cytokines, chemokines, and growth factors, including IFN-γ, TNF-α, granulocyte–macrophage colony-stimulating factor (GM-CSF), IL-10, and IL-13. These factors have been proven to bolster the antitumor immune response and form an unfavorable TME for the tumor [156,208].

## 5. Virotherapy: A Promising Approach to Cancer Treatment

**Figure 4:**
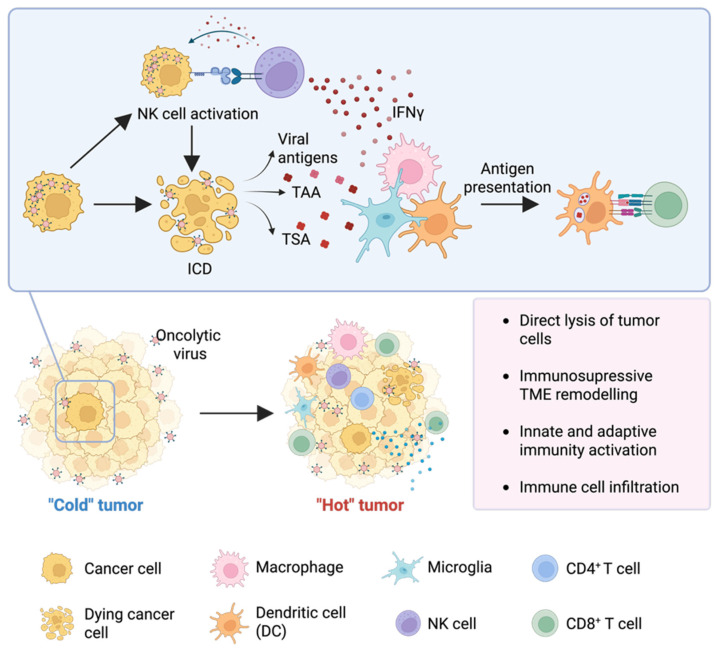
The transformation of the immunologically “cold” tumor microenvironment into a “hot” one achieved through the action of an oncolytic virus. The replication of the oncolytic virus induces the expression of NK cell activating receptor ligands on tumor cells and reduces the expression of inhibitory receptor ligands, increasing the sensitivity of tumor cells to the cytotoxic effects of NK cells. The molecular profile of infected tumor cells optimized for the functional activation of NK cells. Activated NK cells execute a variety of cytotoxic functions through secretion of perforins and granzymes, the production of IFN type I, receptor-mediated cytotoxicity and the induction of apoptosis, that leads to effective elimination of infected tumor cells. During cytolysis, the release of TAA and DAMPs initiates cascading activation of antigen-presenting cells (APC). As these cells migrate to the draining lymph nodes, mature APC perform priming and clonal expansion of tumor-specific cytotoxic T-lymphocytes (CTL). The process of tumor infiltration by activated CTLs to transform an immunosuppressive (“cold”) microenvironment into one is immunogenic (“hot”), thereby inducing a systemic and long-lasting antitumor response.

Virotherapy is regarded as one of the most promising technologies for treating tumors [209,210]. The principal mechanism by which OVs exert their antitumor action is through the direct selective lysis of malignant cells by means of selective viral replication, while sparing healthy cells [211]. The oncoselectivity of viruses is achieved through several mechanisms, including (1) natural selectivity for tumor cells, (2) the use of attenuated vector vaccines (e.g., measles and vaccinia viruses (VV), and (3) genetic modifications of viruses, including deletions of genes required for replication in normal cells, and (4) modification of the expression of proteins interacting with tumor cell receptors. A critical role of OV in tumor therapy is the triggering of ICD, accompanied by the release of damage-associated molecular patterns (DAMPs), such as high mobility group box 1 (HMGB1), adenosine triphosphate (ATP), and calreticulin, pathogen-associated molecular patterns (PAMPs), tumor-associated antigens (TAAs), and tumor-specific antigens (TSAs). These molecular signals induce the production of IFN type I and pro-inflammatory cytokines and chemokines, leading to the activation of DCs, microglia, macrophages, and other antigen-presenting cells. The latter instigates a cascade of activation of specific CD4+ and CD8+ T lymphocytes and NK cells [212]. Activation of innate and adaptive immunity has been demonstrated to transform the immunosuppressive, “cold” TME into an immune-inflammatory, “hot” one, thereby enabling the overcoming of the immunological tolerance characteristic of GBM (Figure 4) [213]. Research has shown that the initial immune response can improve survival rates among GBM patients. Furthermore, OVs have been found to influence stromal cells and ECM components, thereby promoting its degradation and tumor infiltration by T cells [214,215,216].

A wide range of viruses are currently being studied in preclinical studies, including, but not limited to, adenovirus (AdV), herpes simplex virus (HSV) [217], measles virus (MV), parvovirus, poliovirus, retro- and reovirus, Zika virus, Newcastle disease virus (NDV), and VV [218]. However, Delytact (G47∆, Teserpaturev) remains the only viral drug currently approved for the treatment of gliomas. The findings of clinical trials support the favorable safety profile of the utilization of OVs [219]. The development of modified viruses that exhibit enhanced selectivity for tumor cells, augmented replicative capacity, diminished pathogenicity, and heightened immunogenicity is of significant interest [220,221].

### 5.1. Herpesviruses

#### 5.1.1. DL-SPTK

The initial recombinant OV developed for the selective destruction of GBM cells was the herpes simplex virus (dl-sptk). The resultant DLSptk strain exhibited a deletion of the viral thymidine kinase (*tk*) gene, thereby attenuating viral replication in quiescent cells. In preclinical studies, intratumoral injections of dl-sptk demonstrated antitumor activity, as evidenced by the inhibition of tumor growth and the enhancement of survival in experimental animals. However, the deletion of the *tk* gene led to dl-sptk resistance to antiviral drugs targeting this enzyme. This resistance to antiviral therapy has become a significant impediment to its clinical utilization [222].

#### 5.1.2. TVEC

Another prevalent genetic modification detected in the majority of oncolytic HSV-1 strains is a deletion of the neurovirulence gene ICP34.5, which renders the virus unable to replicate in normal cells [223]. In 2015, the European Commission and the FDA approved the use of a genetically modified HSV-1 strain, TVEC (IMLYGIC, talimogene laherparepvec), for the treatment of inoperable melanoma. T-VEC, a recombinant HSV-1 virus with a deletion in the ICP34.5 gene and the ICP47 gene and an insertion encoding GM-CSF, demonstrated an overall response rate of 40.5% in a clinical trial with Imligic, with a median overall survival (mOS) of 41.1%. Favorable outcomes observed in the clinical trials conducted by Imligic have prompted researchers to consider the application of virotherapy in other clinical contexts.

#### 5.1.3. G207

The herpesvirus strain G207 exhibited a favorable therapeutic profile for the treatment of malignant brain tumors [224]. The deletions of both γ34.5 (RL1) loci and the insertion of the lacZ gene into the viral ICP6 (UL39) gene, which inactivates viral ribonucleotide reductase (RR), ensured selective viral replication in actively dividing cells. The insertion of the lacZ marker gene facilitated the histochemical detection of infected cells [224,225]. The safety of G207 was confirmed in a Phase I clinical trial in adult patients with malignant gliomas (NCT03911388) and in a Phase I trial in children with recurrent brain tumors (NCT02457845).

#### 5.1.4. G47Δ

The HSV G47Δ (Teserpaturev or Delytact) was engineered through the introduction of a deletion into the α47 gene of the G207 genome. The α47 deletion in G47Δ led to increased MHC class I expression on infected cells and stimulation of antitumor immune responses. In 2021, the Japan Medicines and Medical Products Agency approved G47Δ for the treatment of brain tumors. In the preliminary Phase I/II investigation (UMIN000002661), the median overall survival (mOS) with two doses of the virus was 7.3 months, and the one-year survival rate was 38.5%. In a phase II clinical trial (UMIN000015995), the mOS for patients diagnosed with GBM after the initiation of therapy was 20.2 months, with a one-year survival rate of 84.2%. A subsequent analysis of tumor biopsies revealed a strong correlation between the dosage regimen and the infiltration of the TME by effector T cells (CD4+ and CD8+). In addition, a decrease in regulatory T cell numbers was observed. G47Δ represents a significant development in the field of cancer research, as it is the first drug since TMZ to demonstrate a positive impact on patient survival. The efficacy of G47Δ has been shown in a number of different types of cancer, including prostate cancer [226], gastric cancer [227], hepatocellular carcinoma [228], tongue cancer [229], esophageal cancer [230], breast cancer [231], neuroblastoma, and malignant peripheral nerve sheath tumors [232]. The administration of OV G47Δ in combination with other treatment modalities has been evidenced to exhibit superior immunotherapeutic efficacy.

#### 5.1.5. rQNestin34.5

In order to enhance the selectivity and cytolytic activity of HSV-1 by reinserting a copy of the γ1 34.5 gene into the viral genome, the rQNestin34.5 strain was constructed. The expression of the ICP34.5 gene was found to be regulated by the Nestin-1 promoter, which has been shown to be specific for GBM cells. In vivo studies found that this strain significantly suppressed tumor growth in comparison to the control group, resulting in a twofold increase in the lifespan of experimental animals [233]. The attenuated HSV-NG34 virus, in which the ICP6 and ICP34.5 genes were deleted, exhibited equivalent efficacy to QNestin34.5.NG34 in mouse models. The absence of ICP6 has been established as a factor that limits oHSV replicative selectivity to mitotic cells or cells with defects in the p16 tumor suppressor, thereby preventing neurovirulence/neurotoxicity in the CNS. ICP34.5 was replaced with its human ortholog GADD34, which was expressed under the transcriptional control of the Nestin cellular promoter/enhancer. NG34 showed high efficacy against GBM in a syngeneic mouse model. Intracerebral administration of NG34 demonstrated a more favorable tolerability profile in immunocompetent and athymic mice in comparison to the parental strain rQNestin34.5 [234].

### 5.2. Adenoviruses

#### 5.2.1. Onyx-015

A substantial proportion of research in the domain of oncolytic virotherapy has been dedicated to AdV. However, the only strain that has undergone clinical trials for GBM remains dl1520 (ONYX-015). The selective oncolytic effect of this strain is achieved through a deletion and mutation in the gene encoding the E1B-55K protein. The E1B protein, which is encoded by the gene, has been observed to suppress p53 activity and prevent apoptosis in the infected cell. In the case of defective E1B, viral replication will only be successful if p53 is defective or absent [235]. The virus was generally well tolerated, but its therapeutic efficacy was found to be limited. The most promising antitumor effects were observed in cases where the drug was administered in combination with chemotherapy [236]. Subsequent to the disclosure of Phase III clinical trial results, Onyx-015 was suspended on the grounds of an absence of effect on mOS [235].

#### 5.2.2. DNX-2401

Is a modified adenovirus of serotype 5. The partial deletion of the E1A gene has been demonstrated to ensure selective viral replication in Rb-deficient tumor cells, while the addition of the RGD peptide has been shown to enhance the virus’s ability to penetrate tumor cells via αvβ3 and αvβ5 integrins. In a Phase I study, it was found that DNX-2401 was both safe and efficacious in terms of its therapeutic effects. Subsequent analyses of tumor samples revealed viral replication and dissemination within the tumor, as well as induction of CD8+ T-cell infiltration, accompanied by a significant reduction in the expression of the T-cell exhaustion marker TIM-3 [237]. A series of clinical trials (NCT01582516, NCT01956734, NCT02798406, NCT02197169) have confirmed the ability of DNX-2401 to induce an effective immune-mediated response against glioma and improve long-term patient survival [52].

#### 5.2.3. Ad-HSV1-TK/GCV and Ad-Flt3L

Preclinical studies further investigated the potential of utilizing a combination of two adenoviral vectors as Ad-HSV1-TK/GCV and Ad-Flt3L [238]. The expression of the FMS-like tyrosine kinase 3 (Flt3L) ligand by the adenoviral vector has been demonstrated to facilitate the recruitment of DCs to the TME. Herpes simplex virus type 1 thymidine kinase (HSV1-TK) has been shown to phosphorylate the prodrug valacyclovir, thereby converting it into a nucleotide analog that induces cell death in actively dividing cells. Adenoviral vectors expressing HSV1-TK/GCV and Flt3L have exhibited encouraging outcomes in preliminary clinical trials. This development subsequently led to the FDA’s approval of a phase I study, which aimed to assess the safety, cell viability, and immunostimulatory efficacy of the treatment in patients diagnosed with high-grade glioma (NCT01811992). In the phase I trial, the treatment was well tolerated, with an mOS of 21.3 months [239].

#### 5.2.4. Poliovirus

PVSRIPO, a live-attenuated vaccine against poliovirus type 1, has a modified ribosome-binding site (IRES). The replacement of the poliovirus IRES with an analogue from human rhinovirus type 2 (HRV2) ensures selective viral replication in neoplastic cells with impaired protein synthesis, while preventing the development of neurotoxicity in normal CNS cells [240]. In 2016, PVSRIPO was awarded Breakthrough Therapy Designation by the FDA, thereby expediting the utilization of genetically modified poliovirus in clinical trials for the treatment of recurrent GBM. A Phase I study (NCT01491893) was conducted to ascertain the safety and efficacy of intratumoral injection of PVSRIPO. The study concluded that the treatment was safe and resulted in a significant survival rate, with approximately 20% of patients remaining alive for 57–70 months following injection. A randomized phase II study (NCT02986178) is currently underway to evaluate the efficacy of PVSRIPO alone and in combination with the alkylating agent lomustine [52].

#### 5.2.5. Parvoviruses

The H-1 parvovirus (H-1PV) has been the subject of research in the context of oncolytic virotherapy for recurrent GBM. This strain is classified as a member of the rat protoparvoviruses and is not known to cause disease in humans. The ParvOryx01 clinical trial (NCT01301430) demonstrated the safety of the administration of the virus, with no adverse events being recorded. While the administration of H-1PV resulted in only a slight increase in mOS, a significant immunostimulatory effect of H-1PV was revealed. A significant increase in the infiltration of cytotoxic CD8+ T lymphocytes was observed in tumors, with a simultaneous decrease in the number of regulatory Tregs [241]. Further substantiation of ParvOryx-mediated immunogenic stimulation was furnished by elevated levels of effector molecules, namely perforin, granzyme B, IFN-γ, IL-2, CD25, and CD40L [231]. H-1PV modulated the immunosuppressed TME, also activating TAM, as evidenced by increased expression of CD68, cathepsin B, and iNOS in patient tumors [241]. The clinical findings indicated that H-1PV possesses considerable promise as a component of combination therapy for recurrent GBM. The combination of H-1PV with bevacizumab (a monoclonal antibody to VEGF) has yielded encouraging results. A documented median survival period of 15.4 months was reported, accompanied by the remission observed in three patients [242].

#### 5.2.6. Reovirus

Reoviruses are naturally occurring OVs that selectively infect tumor cells with an activated Ras signaling pathway. The unmodified Reovirus Type 3 Dearing strain (Reolysin^®^) has shown broad antitumor activity and has been approved by the FDA for the treatment of solid tumors and hematological malignancies [243,244]. Preliminary clinical trials have demonstrated a safe and manageable toxicity profile in patients with GBM (NCT00528684). Its capacity to augment the effectiveness of conventional therapy underscores the promising potential of Reolysin as part of combination regimens [245].

#### 5.2.7. Retroviruses

In the context of developing new treatments for GBM, the retroviral platform implemented in the investigational agent Vocimagene amiretrorepvec (Toca 511) is of interest. This gamma-retroviral vector encodes a cytosine deaminase gene, whose product converts the prodrug 5-fluorocytosine into the cytotoxic drug 5-fluorouracil within cancer cells. In preclinical studies, this strategy enabled high intratumoral production of 5-fluorouracil, depletion of myeloid-derived suppressor cells (MDSCs), and induction of an antitumor immune response [246]. The encouraging results from the preliminary studies (NCT01470794) [240] led to the advancement of Toca 511 to a Phase III clinical trial (NCT02414165). However, results from 2019 indicated that the therapy did not meet its clinically significant efficacy endpoints [247].

#### 5.2.8. Newcastle Disease Virus

NDV is an avian virus that is non-pathogenic to humans and selectively replicates in tumor cells [248]. The oncolytic activity of attenuated NDV strains—HuJ (attenuated by selection) and MTH-68/H (naturally attenuated)—against GBM has been confirmed in preclinical in vitro and in vivo studies, as well as in clinical trials [249]. Treatment with the MTH-68/H strain was associated with marked tumor regression and significant improvements in neurological status [250]. In a dose-escalation clinical trial of the HuJ strain, the median patient survival reached 66 weeks, with one patient achieving a complete response for 3 months prior to subsequent relapse. This oncolytic virotherapy was characterized by exhibiting minimal toxicity alongside clinically meaningful efficacy [251]. The combined application of NDV with TMZ and its poly(lactic-co-glycolic acid) (PLGA) nanoparticle-encapsulated form (TMZ-PLGA-NP) demonstrated a synergistic effect, enhancing antitumor activity in vitro [252].

#### 5.2.9. Vesicular Stomatitis Virus

The findings, based on accumulated data, indicate the capacity of vesicular stomatitis virus (VSV) to directly eliminate tumor cells and to modify the TME, thereby fostering the development of robust antitumor immunity [253]. In order to reduce neurotoxicity and increase the effectiveness of VSV therapy, genetically engineered strains were developed, in particular a chimeric virus (VSVΔG-CHIKV), in which the VSV glycoprotein is replaced by the Chikungunya glycoprotein E3-E2-6K-E1. VSVΔG-CHIKV has been established to be effective in eradicating brain tumors and significantly increasing survival in preclinical models [254]. The chimeric VSV-EBOV virus, in which the VSV glycoprotein has been replaced by the Ebola glycoprotein, demonstrated a favorable safety and efficacy profile. Moreover, the efficacy of the treatment was found to exceed that of the comparison group with respect to the targeting and elimination of brain tumors. In the TME, VSV-EBOV has been observed to increase the number and activity of DC and NK cells, thereby transforming local oncolysis into persistent systemic immunity [255].

#### 5.2.10. The Measles Virus (MV)

The development of MV-based oncolytic virotherapy was informed by clinical data on cancer remission following measles infection [256]. The present study employs live attenuated Edmonston MV strains that exhibit selective tropism for CD46-expressing tumor cells [257]. The oncolytic activity of the Edmonston measles virus (MeV) exerts pleiotropic effects on the antitumor immune response by inducing immunogenic cell death. This, in turn, causes DC activation, antigen presentation, and subsequent T-lymphocyte priming. Furthermore, MeV infection remodels the TME, enhancing both innate (e.g., the repolarization of macrophages and the degranulation of neutrophils) and adaptive antitumor immunity (e.g., the infiltration of T-cells and the CD8+ effector response).

In clinical trials (NCT00390299), the recombinant MV-CEA strain expressing human carcinoembryonic antigen (CEA) demonstrated a favorable safety profile and antitumor activity in patients with recurrent GBM [258]. The combination of MV-CEA with radiation therapy provided a synergistic effect and significantly increased animal survival compared to monotherapy [259]. Recombinant strains expressing the sodium-iodide symporter (MV-NIS), the anti-EGFRvIII antibody (MV-EGFR, MV-EGFRvIII), and the green fluorescent protein (MV-GFP) were developed to improve viral selectivity and facilitate therapy monitoring. MV-NIS and MV-GFP effectively infect and kill glial stem cells both in vitro and in vivo [260]. MV-NIS-based radiovirus therapy exhibited superiority over MV-CEA in the treatment of gliomas [261]. Oncolytic MV strains targeting EGFR/EGFRvIII showed comparable therapeutic efficacy to the unmodified MV-GFP strain [262]. The MV-EGFR therapy induced pro-inflammatory microglia reactivation. A combination strategy incorporating MV-EGFR and immune checkpoint blockade resulted in a significant increase in mouse survival in a syngeneic orthotopic GBM model. Despite the limited replication of the virus in GL261 cells, the therapy exerted a potent immunostimulatory effect, enhancing tumor-targeting CD8+ GzmB+ T cells [263]. Modified MV strains have demonstrated their potential as effective agents in oncolytic virotherapy. However, the large-scale production of highly purified recombinant MeV that complies with good manufacturing practice (GMP) requirements still poses a significant technological challenge [264].

#### 5.2.11. Vaccinia Virus

Vaccinia virus (VV), a double-stranded DNA virus belonging to the Poxviridae family, is one of the most extensively studied viruses worldwide. The combination of natural oncolytic properties, natural tropism for cancer cells, high oncoselectivity, and replicative activity has determined the high potential of VV as a platform for oncolytic virotherapy [265]. Genetic modification enhanced the virus’s selectivity for tumor cells, while concomitantly reducing toxicity and immunogenicity [266,267]. Vaccinia viruses are characterized by their high degree of genetic stability, which facilitates the induction of long-term immune responses, both cellular and humoral [268].

#### 5.2.12. TG6002

TG6002 is a promising recombinant VV strain with potential for the treatment of malignant neoplasms. This particular strain is distinguished by its deletions of the viral *tk* and ribonucleotide reductase (RR) subunit genes, a feature that ensures selective viral replication within tumor cells. A salient feature of the TG6002 genome is the presence of an FCU1 (fusion suicide gene) insertion, which encodes both cytosine deaminase and uracil phosphoribosyltransferase. These enzymes convert the inactive drug 5-fluorocytosine (5-FC) into the cytotoxic agents 5-fluorouracil and 5-fluorouridine monophosphate (5-FUMP) directly in the tumor [269]. A plethora of preclinical studies in vitro and in vivo (on mouse xenografts) have demonstrated the remarkable efficacy and excellent safety profile of TG6002 [270].

#### 5.2.13. LIVP-hIFNα

The recombinant VV strain LIVP-hIFNα, created based on a biovariant of the English Lister (LIVP) strain, exhibited significant toxicity against tumors of various histological origins. This particular strain has a defective *tk* gene and expresses human IFN-α and the fluorescent protein tagRFP. The suppression of viral replication in normal tissues is dependent on the integrity of IFN signaling, thereby ensuring the selective targeting of tumor cells. The immunostimulatory properties of the drug under investigation determine the induction of tumor-specific CTLs, NK cells, macrophages, and the activation of antiangiogenic factors. Preclinical studies have confirmed the efficient replication of the virus in human GBM tumor cell cultures, and the functional activity of IFN expressed by the recombinant LIVP-hIFNα strain [271].

Presently, preclinical studies are investigating the safety profile and therapeutic efficacy of recombinant LIVP variants modified to express immunostimulatory molecules, including IL-15, GM-CSF, anti-PD-1, CCL3, and CCL5 [272].

#### 5.2.14. LIVP-IL-15-RFP

In experiments on orthotopic GBM models, the use of an LIVP strain expressing IL-15 and the fluorescent protein RFP (LIVP-IL-15-RFP) resulted in alterations within the TME. The therapeutic intervention was accompanied by active peritumoral infiltration of CD45+ mononuclear cells, CD206+ macrophages, and stromal elements. Furthermore, viral replication within the tumor elicited a pronounced reactive astroglial response, which spread to virtually the entire ipsilateral hemisphere [272]. The present study investigates the potential of combined approaches based on LIVP strains, as well as animal survival studies. These studies are currently ongoing.

#### 5.2.15. LIVP 1.1.1

In the research of Sunil J. Advani demonstrated that focal ionizing radiation can increase the sensitivity of a tumor to subsequent oncolysis mediated by LIVP 1.1.1, which opens up new prospects for synergy with radiotherapy [273]. However, the effective delivery of the virus to tumor cells remains a key limitation for its systemic use. Immunosuppressive TME, systemic immune barriers and neutralizing antibodies prevent high virus replication in the tumor parenchyma. The solution to this problem was proposed by Elena Ekrami and colleagues. As a “living vector”, they used microglia cells, which are resident immune cells of the brain with high natural migration activity. In in vitro experiments, microglia infected with the LIVP 1.1.1 virus not only successfully supported its replication, but also ensured the effective spread of the virus in three-dimensional models of glioblastoma. In the co-culture system, infected LIVP 1.1.1 microglia facilitated deeper penetration of the virus and resulted in significantly more pronounced oncolysis of tumor cells compared with direct treatment with the viral drug [274].

#### 5.2.16. VV-GMCSF-Lact

The VV-GMCSF-Lact, was developed on the Russian LIVP strain through the deletion of the viral tk and virus growth factor (vgf) genes. Additionally, VV-GMCSF-Lact carries insertions of genes for human granulocyte–macrophage colony-stimulating factor (GM-CSF) and the oncotoxic protein lactaptin, a fragment of human milk kappa-casein [275,276]. The transition to the clinical phase was initiated by the successful completion of preclinical trials, in which VV-GMCSF-Lact demonstrated cytotoxic activity against breast cancer [277,278]. Phase I studies (NCT05376527) in patients with breast cancer, including triple-negative type, have now been completed. Preclinical studies have shown that VV-GMCSF-Lact exerts high levels of cytotoxic activity against glioma cells exhibiting varying degrees of malignancy, particularly against cultures derived from patient samples [279,280]. Furthermore, the capacity to permeate the BBB and the antitumor efficacy was confirmed in a xenograft model of human gliomas and in mouse and rat gliomas in an immunocompetent animal model [281]. The findings from the conducted studies suggest that VV-GMCSF-Lact has the potential to be utilized as a treatment for gliomas.

In summary, the clinical experience with OVs such as G47Δ, DNX-2401, and PVSRIPO has not only confirmed their acceptable safety profile but has also revealed their capacity to induce ICD, remodel the TME, and generate durable antitumor immune responses. These characteristics establish OVs as not standalone “magic bullets”, but rather ideal “partner agents” to sensitize the immunosuppressive GBM TME to other immunotherapies.

## 6. Combination of Oncolytic Virotherapy and Modern Methods of Glioblastoma Immunotherapy

As outlined in Section 5, OVs have been demonstrated to be effective inducers of immunogenic cell death and can remodel the immunosuppressive TME, effectively “warming up” cold tumors. This fundamental property renders them optimal collaborators in combination with other immunotherapies, such as immune checkpoint inhibitors and adoptive cell therapies, to surmount the limitations of GBM treatment (Table 1).

### 6.1. Virotherapy in Combination with ICI

To date, the use of OV with ICI is the most studied area of combination immunotherapy for glioblastoma [237]. The multicenter CAPTIVE II phase study (NCT02798406) confirmed the safety and efficacy of this approach. The clinical efficacy of the treatment was attributable to the local effect of OB on the TME and systemic activation of innate and adaptive immunity. The PD-1 blockade has been shown to neutralize immunosuppression that is mediated by immune checkpoints. In a group of 42 patients, the median overall survival reached 12.5 months, with 20.2% of patients surviving beyond 18 months. An objective response or stabilization of the disease was observed in 56.2% of patients, with three of these patients exhibiting sustained responses to therapy at 45, 48 and 60 months, respectively. The data obtained indicated a favorable safety profile for the combined approach and the possibility of long-term control of glioblastoma [303].

Research conducted by Zineb Belcaid and colleagues has demonstrated that infection with the Delta24-RGD virus affects pivotal immune regulatory factors, leading to an increases the expression of PD-L1 by tumor cells and PD-1 by CD8^+^ T cells. The combination of low doses of OV with PD-1 antibodies resulted in a significant increase in survival in syngeneic mouse models of glioma compared to therapy with each agent alone. This approach overcame immune resistance associated with PD-1-positive CD8^+^ T cells and activated a potent IFNγ-dependent antitumor immune response [282]. Another regimen demonstrated similar efficacy. The combination of ICOVIR17 with ICI resulted in pro-inflammatory polarization of macrophages and specific T-cell cytotoxicity. The result was a prolonged period of remission in animals with GBM [215]. The combination of reovirus with anti-PD-1 therapy enhanced antitumor activity against GBM by regulating the PD-1/PD-L1 axis and activating cytotoxic T lymphocytes [283]. The combination of H-1PV virotherapy with nivolumab and bevacizumab provided an objective response in 78% of patients with glioblastoma (7 out of 9), which was more effective than bevacizumab and ICI alone [305]. The combination of PVSRIPO with PD1/PD-L1 blockade has been shown to promote tumor growth suppression and long-term antitumor activity [306].

The recombinant oncolytic vaccine virus VVLΔTKSTCΔN1L-mIL2 has emerged as a promising candidate for combination with ICI. Its combination with anti-PD1 antibodies caused the TME remodeling, including reprogramming of macrophages to the pro-inflammatory M1 phenotype, increased NK cell infiltration of the tumor, and increased numbers of effector CD4+ and CD8+ T cells and memory T cells. This culminated in the establishment of a protracted antitumor immune response. In glioma models, 80% of mice were cured, and no relapses were observed during the 180-day observation period [284]. The enhancement of immunotherapy effects was also characteristic of the combination of G47Δ with an antibody to CTLA-4. This therapy regimen induced a “warming” (or immunomodulatory) effect on the “cold” TME, thereby promoting active recruitment of effector T cells and concomitant reduction of the Treg population. The combination of these two therapeutic modalities has been shown to promote the development of potent adaptive antitumor immunity [286]. Furthermore, the Herpes simplex virus OV G47∆ strain, harboring a mouse IL-12 insert (G47∆-mIL12), was examined in conjunction with antibodies targeting PD-1 and CTLA-4. The triple therapy enhanced CD4+ and CD8+ T-cell infiltration of the tumor while depleting the pool of immunosuppressive Tregs. As a result, the long-term survival rate was found to be 89%, in comparison to 37% in the ICI alone group. Furthermore, the cured mice developed specific immunological memory, as evidenced by the absence of tumor development following the re-implantation of cancer cells [287]. A herpes simplex virus strain, M032, expressing human IL-12, is currently undergoing clinical trials (NCT05084430). It is being evaluated as part of a combination therapy with the pembrolizumab for the treatment of recurrent malignant glioma.

The development of viruses that directly express antibodies to PD-1 has marked a new stage in the development of a combination approach using ICIs. In preclinical studies, the modified C5252 virus, expressing IL-12 and anti-PD-1 antibodies, provided significant suppression of tumor growth and increased the lifespan of experimental animals [285]. The accumulated data suggest that a combined strategy of virotherapy with ICI is promising and highly effective for the treatment of glioblastoma, a tumor with a “cold” immune phenotype.

### 6.2. Virotherapy in Combination with Adoptive Cell Therapy

The capacity of OVs to act as inducers of antitumor immune responses provides the fundamental basis for their successful use in combination with adaptive immunotherapy. On the other hand, OVs have been shown to enhance the visibility of tumors to the immune system, thereby reversing immunosuppression within the TME. This, in turn, fosters the effective functionality of CAR cells. The complementary mechanisms of action enable the circumvention of the limitations inherent to each agent thereby facilitating the attainment of a more pronounced clinical effect.

A recent study that combined oncolytic adenovirus expressing CXCR11 and B7H3-CAR-T cells revealed that the administration of CXCR11-oAd led to the effective destruction of cancer cells. In addition to this primary finding, the study demonstrated that the treatment also enhanced the recruitment of CAR-T cells to the tumor site and rejuvenated the TME, which had previously been characterized as “cold”. In GBM models, there was an increase in the infiltration of CD8+ T lymphocytes, NK cells, and M1-polarized macrophages, while the proportion MDSC, Treg cells, and M2-polarized macrophages decreased. CAR-T cells targeting B7H3 showed no capacity to inhibit GBM growth when administered alone. However, when delivered intratumorally in conjunction with the CXCL11-oAd, the CAR-T cells elicited a sustained antitumor response [289]. The combination of B7H3-CAR-T therapy with other treatments increased T-cell proliferation and significantly improved antitumor efficacy in comparison with either OV or CAR-T-cell therapy administered alone. In addition, oncolytic adenovirus (oAd-IL17) with an insert of interleukin-17 stimulated the proliferation and survival of tumor-infiltrating B7H3-CAR-T cells; however, it did not reverse their exhaustion [290]. The combination therapy involving CD70-specific CAR-T cells and oHSV-1 resulted in the elimination of tumors by means of enhancing pro-inflammatory processes and reducing anti-inflammatory factors. Furthermore, therapeutic intervention led to a modification in the immune profile of the tumor, characterized by an increase in the proportion of T and NK cells and a concomitant decrease in Tregs and TGFβ levels. By releasing IFN-γ and stimulating T-cell infiltration into the tumor, oHSV-1 enhanced the therapeutic efficacy of CD70-specific CAR-T cells [292]. Conte’s study demonstrated that the combination of oHSV with IL-13Rα2-CAR-T cells resulted in significantly enhanced tumor cell destruction compared to the use of either monotherapy. The findings of this study, as indicated by mathematical modeling, underscore the pivotal role of the sequence of therapy in this context. To optimize the therapeutic benefits of this approach, it is recommended that the administration of OV and CAR-T cells be conducted in a synchronized manner, either concurrently or with OV preceding cell therapy. A time interval exceeding 24 h between OV and CAR-T cells led to a substantial diminution in the therapeutic effect, underscoring the necessity for temporal synchronization of therapeutic components [294]. In contrast to the conventional approach of administering therapeutic agents individually, an integrated delivery strategy employing OV CAR-T cells was proposed. B7H3-CAR-T effectively delivered HSV to the tumor, resulting in local effects in the tumor, enhanced T-cell infiltration, and a significant increase in the survival of experimental animals [291]. Similarly, by incorporating OAd encoding IL15 into the CAR-T platform, it was possible to achieve precise targeting and intratumoral release of the virus. This design enhanced viral oncolysis, increased T-cell proliferation, persistence, and cytolytic activity, optimized the co-localization of therapeutic agents, and maintained CAR-T cell functionality through IL15. This approach made it possible to overcome the therapeutic resistance of glioblastoma and increase antitumor efficacy [293].

The employment of OV and CAR-NK cells in a therapeutic context constitutes an additional promising combination therapy strategy. The latter have been shown to exhibit certain advantages over CAR-T cells, including a reduced risk of adverse effects and a potentially enhanced capacity to penetrate brain tissue. Rui Ma and colleagues utilized an OV based on HSV-1 expressing human IL15/IL15Rα with a “sush” domain (OV-IL15C), in conjunction with pre-existing EGFR-CAR-NK cells, to assess their efficacy under various treatment regimens. The therapeutic intervention led to an augmentation in intracranial infiltration and activation of NK and CD8+ T cells, accompanied by an enhancement in the persistence of CAR-NK cells within an immunocompetent model. The immunomodulatory effect of the drug was found to effectively suppress tumor growth and significantly increase the median overall survival of animals [295].

The research conducted by Carter M. Suryadevara and his colleagues examined the mechanism of synergy of immunotherapeutic approaches based on the characteristics of immunogenic death of tumor cells. Bortezomib therapy (a proteasome inhibitor) led to tumor necroptosis and increased oHSV proliferation in infected glioma cells. The release of pro-inflammatory factors by necrotic cells resulted in increased activation of NK cells and the production of effector cytokines. The combination of these two modalities resulted in enhanced immune cell involvement and increased sensitivity of infected cancer cells to NK cell-mediated destruction [296,297]. Haoran Du and colleagues have proposed an innovative approach that incorporates dual targeting capabilities. CAR-NK cells were engineered to target IL13R2 and CD19, as well as to include IL6 and IL21. The OV HSV-1 was engineered to express CD19 and chemokine CCL5. This helped attract NK cells and target the tumor. Consequently, combination therapy resulted in substantial tumor regression, augmented immune cell infiltration, and increased survival outcomes when compared to monotherapy [298].

Despite the necessity for additional research to optimize delivery regimens and select the most effective virus and CAR cell designs, combined strategies have the potential to pave the way for the creation of effective synergistic therapeutic programs for the treatment of GBM. The findings from ongoing studies offer substantial experimental evidence supporting the potential implementation of combination immunotherapy in future clinical practice for GBM.

## 7. Discussion and Future Directions

High-grade gliomas (HGGs) are the most aggressive type of CNS tumor. Conventional therapeutic modalities, encompassing tumor resection, chemotherapy, and radiation therapy, have demonstrated limited therapeutic efficacy, with median patient survival rates remaining low. The TME is a key factor in the ineffectiveness of today’s treatments because it suppresses antitumor immunity, allowing tumors to grow and spread. Scientific data show that immunotherapy can counteract this suppression and enhance antitumor immunity, improving patient response to therapy.

The employment of checkpoint inhibitors, CAR-T cells, NK cells, and OVs holds considerable potential for the treatment of immunologically complex “cold” tumors, such as HGGs. The most promising therapeutic approach is to shift treatment strategies from monotherapy to complex combination approaches (Table 1). The synergistic mechanisms of action inherent to each constituent of combination therapy significantly enhance treatment efficacy. The utilization of OVs, which infect tumor cells with impaired DNA repair mechanisms, has been demonstrated to potentiate the cytotoxic effects of chemotherapy. In addition, chemotherapeutic agents have been shown to markedly augment the replication of OVs [307]. Pre-treatment with cyclophosphamide during oHSV-G47∆ virotherapy resulted in enhanced tumor cell lysis and increased treatment efficacy [308]. The combination of Delta24-RGD adenovirus and TMZ exhibited a synergistic cytotoxic effect [309]. The combination of virotherapy and radiation holds promise as a treatment for gliomas, given the capacity of viruses to augment radiation-induced tumor cell apoptosis. The clinical significance of the efficacy of the oncolytic HSV-1 virus, G207, when used in combination with radiation therapy has been confirmed [310]. Research employing AdvHSV-tk in conjunction with ionizing radiation has yielded encouraging outcomes, as evidenced by the findings reported in reference [311]. The concomitant administration of oncolytic virotherapy and checkpoint inhibitors was found to result in a favorable safety profile, as well as the potential to achieve long-term disease control. Intratumoral administration of DNX-2401 followed by pembrolizumab therapy resulted in a significant improvement in patient survival [303]. The capacity of OVs to stimulate antitumor immunity by enhancing tumor infiltration with T lymphocytes has been demonstrated to significantly increase the efficacy of adoptive cell therapy. A study of an CXCL11-adenovirus expressing chemokine (CXCL11-oAd), in combination with anti-B7H3 CAR-T cells, revealed enhanced CAR-T cell recruitment to the tumor and reprogramming of the immunosuppressive TME [301].

A significant challenge of modern therapy is the presence of dormant cancer cells with reduced metabolism resistant to chemotherapy and responsible for the recurrence of cancer [312]. Moreover, the possibility of reactivation of these cells after a course of therapy aimed at heating TME was shown. Within the framework of the modern concept, the plasticity of tumor cells is an important hallmark of cancer, including the ability of cells under stress (exposure to therapeutic agents) to go into a less active dormant state with no proliferation, as well as later reactivate with the resumption of the ability to metastasis and tumor growth [313]. For example, bleomycin-induced lung inflammation provokes the release of tumor cells from the state of dormancy [314].

Overcoming this dormant reservoir is a fourth frontier in GBM immunotherapy, alongside TME remodeling, antigen heterogeneity, and immune exhaustion. A range of strategies are being developed to address this challenge. First, combination therapies must be designed to specifically target GSCs, which are the primary architects of dormancy. One particularly promising avenue of research involves the use of OVs, which have been engineered to selectively replicate and lyse stem-like tumor cells, irrespective of their proliferative status [315]. Secondly, therapeutic regimens must incorporate agents capable of “awakening” dormant cells, thereby inducing them to exit a state of quiescence and enter a vulnerable state susceptible to immune attack or chemotherapy. Cytokines such as IFN-α and TNF-α, or even components of the inflammatory milieu induced by OV infection, have been shown to disrupt dormancy programs [316]. Thirdly, the maintenance of prolonged immune surveillance is of the utmost importance. The objective is not only the eradication of the primary tumor mass, but also the establishment of a persistent, systemic memory response that is capable of continuously monitoring and eliminating any cell that attempts to emerge from dormancy. This objective can be accomplished through the administration of vaccines, such as dendritic cell vaccines pulsed with tumor antigens, in the adjuvant setting. Alternatively, the use of OVs armed with immune-stimulatory cytokines, including IL-12 and IL-15, has been demonstrated to promote memory T-cell formation [317]. The combination of G47∆-mIL12 with checkpoint inhibitors, which resulted in long-term survival and protective immunological memory in murine models, exemplifies this approach [287].

The most promising approach is the combination of OV with modified NK cells, specifically CAR-NK cells. A strategy employing the oncolytic HSV expressing IL-15/IL-15Rα (OV-IL15C) in conjunction with EGFR-CAR-NK cells resulted in a synergistic suppression of tumor growth and a significant increase in survival in experimental animals. The OV-IL15C treatment has been demonstrated to promote intratumoral infiltration of systemically administered CAR-NK cells, thereby increasing their persistence and cytotoxic activity within the tumor [174,295]. The combination of bortezomib, an inhibitor of the proteasome, with an oncolytic HSV and NK cells, known as triple therapy, exhibited a synergistic antitumor effect in GBM. Bortezomib enhances viral replication and induces necrosis, which releases pro-inflammatory factors that activate NK cells and produce effector cytokines, killing tumors [296]. The combination of immunotherapy strategies and OVs represents a promising approach to GBM therapy, with the potential to overcome the limitations of monotherapy regimens and achieve clinical success in the treatment of this complex malignancy.

However, despite the impressive successes and encouraging results, combination therapy for GBM using OVs currently faces a number of serious limitations and obstacles. The centralization of addressing these issues in contemporary research is imperative for enhancing the clinical efficacy of GBM therapy. A significant challenge pertains to the absence of optimal solutions for drug combinations, dosages, dosing regimens for each component, their sequence, and routes of administration. The utilization of high-throughput testing platforms in a systematic screening approach is imperative for the optimization and identification of the most efficacious combinations. It is imperative that dosing regimens are tailored to individual tumor susceptibility, as well as the results of extensive safety studies and dose–response studies of combination therapy. The sequence of drug administration should be based on preclinical studies of time windows, with identification of critical points for synergy [300]. Moreover, the establishment of suitable response criteria is imperative. Presently, standard treatment evaluation criteria are not applicable to immunotherapy due to pseudo-progression, whereby tumor size increases not due to tumor growth but to massive immune cell infiltration. In order to achieve a more accurate assessment of the therapeutic effect, it is necessary to establish new, more adequate criteria and to utilize functional and metabolic PET scanning.

Sonoporation can facilitate the penetration of the BBB by systemically administered agents such as CAR-T/NK cells and OB. Convection-Enhanced Delivery (CED) provides high, uniform distribution drug concentrations in tumors while minimizing systemic side effects and saving therapeutic activity (NCT01491893 PVSRIPO). For short-lived agents (e.g., CAR-T, NK cells, OVs), implantable delivery systems enable repeated local dosing, maintaining therapeutic levels until a complete response is achieved [318].

Innovative delivery approaches, including nanoparticles (NPs), and the use of living cells as Trojan horses overcome the limitations of systemic delivery. NPs can bring an advantage by either passive enhanced permeability and retention or by active targeting to tumor cells using specific ligands [319,320]. For example, NPs containing the recombinant enterovirus EV-A71-miR124T cross the BBB, causing glioma regression and prolonged survival in vivo [321]. Alternatively, delivering viral genetic material as part of NPs, rather than the entire virus. The method circumvents issues associated with the manufacturing and stability of live viruses, employing pre-approved and scalable platforms such as lipid nanoparticles (LNPs). NP-vGenome has been shown to retain its oncolytic activity even in the presence of neutralizing antibodies, and it has the capacity to elicit a robust antitumor immune response [320]. Furthermore, living cells have emerged as a promising platform for the delivery of OV. Neural and mesenchymal stem cells exhibit a natural tropism for tumor cells, which, when administered systemically, enables them to effectively deliver therapeutic agents, thereby masking them from the immune system (NCT03072134, NCT03896568) [322,323].

A further significant challenge pertains to the high heterogeneity exhibited by tumor cells and the TME. Concurrent targeting of multiple antigens has been demonstrated to enhance the probability of complete eradication of a heterogeneous tumor, whilst concomitantly preventing the tumor from evading therapy. The identification of new biomarkers specific to GBM facilitates the development of targeted OVs, CAR-T and CAR-NK cells that target the unique molecular features of the tumor. Advancements in this domain hold promise for the identification of novel cell surface antigens as targets for personalized antitumor therapy [283,324,325].

The development of platforms that regulate the expression of immunomodulators (e.g., IL-12, IL-15, IL-21, GM-CSF, or CCL5) facilitates the overcoming of the immunosuppressive barrier characteristic of GBM, thereby enhancing the efficacy of cell and viral therapies. Multitargeted combination therapy is imperative for overcoming the immunosuppression inherent to the complex and highly adaptive nature of GBM. In this context, the utilization of preclinical models that reflect the heterogeneous characteristics of the tumor and its TME is imperative for comprehending the effects of combination therapy. Heterotypic 3D models consisting of multiple cell types provide an excellent model for mimicking tumor heterogeneity and its physiology [326]. The study of tumor models that maximally preserve the molecular biological characteristics of the primary tumor (personalized cell cultures) and that are capable of simulating interactions between the tumor, OVs, NK cells, and other components of the immune response and the TME. The utilization of 3D models has the potential to facilitate the development of novel therapeutic approaches that target both the tumor and the TME. The hypothesis posits that the development of combination therapy strategies employing 3D models in preclinical studies holds promise for achieving breakthroughs in the treatment of malignant gliomas. It is essential that these 3D models incorporate the biology of dormancy, for example, by generating hypoxic or nutrient-deprived niches that promote GSC quiescence, to accurately screen for therapies capable of eradicating the dormant reservoir [314]. It is imperative to acknowledge the significance of integrating dormancy as a pivotal variable within the framework of preclinical testing. This approach is essential for the development of combination regimens that aspire not merely to achieve remission, but rather to attain a lasting cure.

## 8. Conclusions

A review of the extant literature reveals noteworthy advancements in the domain of GBM immunotherapy, accompanied by encouraging outcomes and substantiated clinical efficacy [12,327,328,329]. The array of strategies being developed is indicative of the intricate nature of the disease, which is marked by significant heterogeneity and substantial resistance to treatment.

At the core of this challenge lies the profound immunosuppressive tumor microenvironment (TME) that renders GBM one of the most formidable “cold” tumors. The primary strategic imperative, therefore, is not merely to kill tumor cells, but to fundamentally rewire this microenvironment—to overcome immune tolerance and establish a durable, systemic antitumor response. The therapeutic modalities discussed herein represent distinct yet complementary tools for achieving this goal. Checkpoint inhibitors reactivate the T-cell response by removing the brakes on immunity, while CAR-T and NK cells provide targeted cytotoxicity as effector agents. Crucially, oncolytic viruses combine direct lysis of tumor cells with a powerful induction of immunogenic cell death, effectively serving as the “warming-up” agents that can convert the immunological desert of GBM into an inflamed, recognizable battlefield.

The clinical reality indicates that these approaches alone have not demonstrated sufficient efficacy to achieve a lasting cure. Despite the effectiveness and proven therapeutic potential of each of these approaches, achieving a clinically significant effect in GBM remains limited. This review has synthesized the growing body of evidence that the future of GBM therapy lies in the rational combination of these modalities. The most compelling data emerge from the synergy between OVs and adoptive cell therapies, particularly with NK cells.

However, translating these promising pre-clinical synergies into clinical areas will require addressing several critical translational and regulatory challenges. Firstly, it is imperative to emphasize the necessity of the development of standardized, “off-the-shelf” allogeneic CAR-therapy. This is essential in order to overcome the logistical and cost-related barriers that currently limit the accessibility of combination therapies to a broader patient population. Secondly, the design of clinical trials must evolve to accommodate the unique kinetics of combination immunotherapy. It is imperative that traditional response criteria be meticulously implemented to differentiate authentic progression from pseudo-progression, a phenomenon that is especially evident following OV therapy. The employment of adaptive trial designs, incorporating biomarker-driven stratification, will be pivotal in identifying patient subsets that exhibit a high response rate to specific combinations of OV + cell therapy, as defined by tumor mutational burden, baseline immune infiltration, or specific antigen expression. Thirdly, the optimal sequencing, dosing, and route of administration (intratumoral, intraventricular, or intravenous with BBB disruption) must be empirically defined through carefully designed Phase I/II studies, moving beyond empirical combinations to rationally designed regimens. A critical bottleneck in OV research is the paucity of large, randomized clinical trials, particularly Phase III/IV studies that evaluate long-term safety, survival benefits, and quality-of-life outcomes. The majority of extant trials comprise small patient cohorts with heavily pre-treated or refractory disease, which renders generalization difficult. The clinical adoption of these agents is further delayed by recruitment challenges, financial constraints and regulatory complexities [330].

The development of combined treatment regimens that take into account both the complex and adaptive nature of GBM and the features of its heterogeneous immune-suppressive TME seems to be a prerequisite for achieving a breakthrough in the treatment of this malignant disease. In order to achieve clinical success in the future, it is essential to address the critical challenges outlined in this review. This involves transitioning from empirical combinations to rationally designed, multi-modal regimens guided by advanced 3D preclinical models. Innovative solutions are imperative for crossing the blood–brain barrier; these include the use of cellular “Trojan horses” and convection-enhanced delivery. A critical aspect of this approach involves a transition towards personalized strategies that target multiple antigens in a simultaneous manner, with the objective of effectively overcoming the heterogeneity characteristic of tumors.

In summary, it is evident that a single, effective solution to the problem of GBM will not be found. The future of cancer treatment is predicted to be marked by the integration of various therapeutic modalities. Multi-targeting GBM with drug combinations represents the future directions, where refined design principles guided by the contemporary understanding of “cancer hallmark co-targeting” could enhance therapeutic impact.

## Figures and Tables

**Table 1:** Combined approaches to virotherapy and adoptive cellular therapy in glioblastoma.

Study Type	Oncolytic Virus	Combination Therapy	Outcome	Reference
PCS	ICOVIR17	α-PD1	Induction of pro-inflammatory activity of TAM and tumor-specific cytotoxicity of T cells, achievement of long-term remission, increase in OS	[215]
PCS	Delta24-RGD	α-PD1	Increased in TME CD8+ T cells, increased IFNγ level, increased mOS	[282]
PCS	Reolysin	α-PD1	Increased cytotoxic T cells in TME, enhanced antitumor response, magnification OS	[283]
PCS	VVΔTK-STCΔN1L-mIL-21	α-PD1	80% cure and no relapses during the 180-day follow-up period	[284]
PCS	C5252	α-PD1	Increased antitumor activity, inhibition of GBM progression, statistically significant increase in mOS	[285]
PCS	G47Δ	α-PD1	Efficacy equivalent to anti-PD-1 monotherapy	[286]
PCS	G47Δ	aCTLA-4	Increased antitumor activity, increased mOS, 5 out of 8 animals have achieved a cure	[286]
PCS	G47Δ-mIL-12	α-PD1 aCTLA-4	The survival rate was 89%, 5 out of 7 animals have achieved a cure, increased number of Teff, TAM type1, decreased number of Treg	[287]
PCS	XVir-N-31	α-PD1 (nivolumab)	Increase in the number of TILs and NK cells, decrease in the growth of contralateral tumors	[288]
PCS	CXCL11-oAd	B7H3-CAR-T cells	Enhanced recruitment of CAR-T cells to the tumor and reprogramming of immunosuppressive TME	[289]
PCS	oAD-IL7	B7H3-CAR-T cells	Improved proliferation and persistence of B7H3-CAR-T in the TME increased mOS of experimental animals	[290]
PCS	HSV	B7H3-CAR-T cells	Improving the efficiency and bio-distribution profile of OV increases the mOS of experimental animals	[291]
PCS	oHSV-1	CD70-CAR-T cells	Increased TME infiltration of CD4+ and CD8+ T-cells, decrease in Treg, disappearance of tumors in mice by day 70 of observation, increase in mOS	[292]
PCS	OAd	CD70-CAR-T- cells	Enhanced viral oncolysis, increased T cell viability, reduced immunosuppression in the TME, and high antitumor efficacy both in vitro and in vivo	[293]
PCS	C134	IL-13Rα2- CAR-Tcells	Effective destruction of tumor cells	[294]
PCS	OV-IL15C	EGFR-CAR NK cells	Enhanced infiltration and activation of NK and CD8+ T cells in the TME, increased CAR-NK cell persistence, tumor growth suppression, increased mOS	[295]
PCS	HSV-1	proteasome inhibitor (bortezomib), NK cell	Necroptosis induction, enhanced NK cell activation, improved tumor elimination	[296]
PCS	HSV-1	proteasome inhibitor (bortezomib), NK cell	Increased activation of NK cells, increased antitumor efficacy.	[297]
PCS	HSV-1	IL13Ra2 and CD19-CAR-NK cells	An increase in NK cell infiltration and persistence in the TME is expected.	[298]
PCS	G47Δ	Lp2-CAR-T cells	Tumor regression, increased mOS	[299]
PCS	G207	BiTE	Increase in specific cytotoxicity	[300]
PCS	HSV-1	NK cells	Elimination of OV, oHSV clearance was 80%	[301]
PCS	oHSV	TGF-β1	Temporary suppression of innate immune response, increased effectiveness of oHSV therapy, reduced tumor growth, increased mOS	[302]
Phase I	Ad-RTS-hIL-12	veledimex, α-PD1 (nivolumab)	mOS 16.9 months	NCT03636477
Phase I	Reovirus wild-type	GM-CSF (sargramostim)	N/A	NCT02444546
Phase I b	DNX-2401	IFN-γ	OS-12–33%, OS-18–22%, three patients survived 19, 21 and 22 months	NCT02197169
Phase I/II	DNX-2401	α-PD1 (pembrolizumab)	mOS 12.5 months, OS 18–20.2%	NCT02798406 [303]
Phase I/II	M032	α-PD1 (pembrolizumab)	N/A	NCT05084430
Phase I/II	PVSRIPO	α-PD1 (atezolizumab)	N/A	NCT03973879
Phase II	PVSRIPO	α-PD1 (pembrolizumab)	mOS 10.2 months	NCT04479241
N/A	H-1PV	ICI, anti-VEGF (bevacizumab)	Objective response to therapy in 7 out of 9 patients (78%). Two patients had a complete response, 5 patients had partial remission, 2 patients had disease progression	[304]

B7-H3 (CD276) = a member of the B7 family of proteins, CAR = chimeric antigen receptor, EGFR = epidermal growth factor receptor, GBM = glioblastoma, GD2 = disialoganglioside, GMCSF = granulocyte–macrophage colony-stimulating factor, HSV = Herpes simplex virus, ICI = immune checkpoint inhibitors, mOS = median overall survival, N/A = not available, NK = natural killer, OS = overall survival, OV = oncolytic virus, PCS = preclinical studies, TAM = tumor-associated macrophages, Teff = T effector cells, TGF = transforming growth factor, TILs-tumor-infiltrating lymphocytes, TME = tumor microenvironment, VEGF = vascular endothelial growth factor.

## Data Availability

No new data were created or analyzed in this study.

## Supplementary Materials

The following supporting information can be downloaded at: https://www.mdpi.com/article/10.3390/ijms27052457/s1.

## Abbreviations

The following abbreviations are used in this manuscript:

ADCC	antibody-dependent cell-mediated cytotoxicity
Adv	adenovirus
Arg-1	arginase-1
ATP	adenosine triphosphate
BBB	blood–brain barrier
bFGF	basic fibroblast growth factor
B7-H3 (CD276)	a member of the B7 family of proteins
CAF	cancer-associated fibroblasts
CAIX	carbonic anhydrase IX
CAR	chimeric antigen receptor
CEA	carcinoembryonic antigen
CED	convection-enhanced delivery
CNS	central nervous system
CTLA-4	cytotoxic T-lymphocyte-associated protein 4
CTLs	cytotoxic T lymphocytes
CRS	cytokine release syndrome
CSPG4	chondroitin sulfate proteoglycan 4
DAMPs	damage-associated molecular patterns
DCs	dendritic cells
DIPG	diffuse intrinsic pontine glioma
DLT	dose-limiting toxicity
EGFR/ErbB	epidermal growth factor receptor
EGFRvIII	epidermal growth factor receptor variant III
ECM	extracellular matrix
EphA2	erythropoietin-producing hepatocellular A2
GAL-9	galectin-9
GBM	glioblastoma
GD2	disialoganglioside
GMCSF	granulocyte–macrophage colony-stimulating factor
GSCs	glioma stem cells
HER2	human epidermal growth factor receptor 2
HGG	high-grade glioma
HMGB1	high mobility group box 1
HLA	human leukocyte antigen
HSV	Herpes simplex virus
ICI	immune checkpoint inhibitors
IHC	immunohistochemistry
iNOS	nitric oxide synthase
iPSC	induced pluripotent stem cells
KIR	killer-cell immunoglobulin-like receptors
LAG-3	lymphocyte activation protein 3
LAK	lymphokine-activated killer cells
LNPs	lipid nanoparticles
MDSC	myeloid suppressor cells
MeV	Edmonston measles virus
MHC	major histocompatibility complex
MMPs	matrix metalloproteinases
mOS	median overall survival
MV	measles virus
N/A	not available
NPs	nanoparticles
NCR	natural cytotoxicity receptors
NDV	Newcastle disease virus
NK	natural killer
NKG2D	integral membrane protein type II
OS	overall survival
OV	oncolytic virus
PAMPs	pathogen-associated molecular patterns
PD-1	programmed cell death protein 1
PD-L1	programmed death-ligand 1
PLGA	poly(lactic-co-glycolic acid)
PTEN	phosphatase and tensin homolog deleted on chromosome 10
ROS	reactive oxygen species
STI1	stress-induced protein 1
TAAs	tumor-associated antigens
TAM	tumor-associated macrophages
TGF	transforming growth factor
Th	T-helpers
TILs	tumor-infiltrating lymphocytes
TIM-3	T-cell immunoglobulin and mucin-containing domain-3
TIME	tumor immunophenotypes
*tk*	thymidine kinase
TMB	tumor mutational burden
TME	tumor microenvironment
TNF	tumor necrosis factor
TMZ	temozolomide
Tregs	T-regulatory cells
TSAs	tumor-specific antigens
VEGF	vascular endothelial growth factor
VDX	Veledimex
*vgf*	virus growth factor
VSV	vesicular stomatitis virus
VV	vaccinia virus
