# Supplementary material for: Heat up and Destroy: Immunotherapy of “Cold” Tumors Using the Example of Glioblastoma
Source: Int J Mol Sci. 2026 Mar 7;27(5):2457. doi: 10.3390/ijms27052457 (PMC12985887; doi:10.3390/ijms27052457)
Supplement: Supplementary file 1 [file ijms-27-02457-s001.zip › ijms-4143104-supplementary.pdf]

International Journal of
Molecular Sciences

MDPI

Supplementary Materials

Table S1. Functional characteristics of the targets obtained using String.

Gene	Function
Arg1	Arginase-1; Key element of the urea cycle converting L-arginine to urea and L-ornithine, which is further metabolized into metabolites proline and polyamides that drive collagen synthesis and bioenergetic pathways critical for cell proliferation, respectively; the urea cycle takes place primarily in the liver and, to a lesser extent, in the kidneys. Belongs to the arginase family.
iNOS	Nitric oxide synthase, inducible; Produces nitric oxide (NO) which is a messenger molecule with diverse functions throughout the body. In macrophages, NO mediates tumoricidal and bactericidal actions. Also has nitrosylase activity and mediates cysteine S-nitrosylation of cytoplasmic target proteins such PTGS2/COX2.
B7-H3	CD276 antigen; May participate in the regulation of T-cell-mediated immune response. May play a protective role in tumor cells by inhibiting natural-killer mediated cell lysis as well as a role of marker for detection of neuroblastoma cells. May be involved in the development of acute and chronic transplant rejection and in the regulation of lymphocytic activity at mucosal surfaces. Could also play a key role in providing the placenta and fetus with a suitable immunological environment throughout pregnancy.
CTLA-4	Cytotoxic T-lymphocyte protein 4; Inhibitory receptor acting as a major negative regulator of T-cell responses. The affinity of CTLA4 for its natural B7 family ligands, CD80 and CD86, is considerably stronger than the affinity of their cognate stimulatory co-receptor CD28.
LAG-3	Secreted lymphocyte activation gene 3 protein; Lymphocyte activation gene 3 protein: Inhibitory receptor on antigen activated T-cells. Delivers inhibitory signals upon binding to ligands, such as FGL1 (By similarity). FGL1 constitutes a major ligand of LAG3 and is responsible for LAG3 T-cell inhibitory function (By similarity). Following TCR engagement, LAG3 associates with CD3-TCR in the immunological synapse and directly inhibits T-cell activation.
PD-1	Programmed cell death protein 1; Inhibitory receptor on antigen activated T-cells that plays a critical role in induction and maintenance of immune tolerance to self. Delivers inhibitory signals upon binding to ligands CD274/PDCD1L1 and CD273/PDCD1LG2. Following T-cell receptor (TCR) engagement, PDCD1 associates with CD3-TCR in the immunological synapse and directly inhibits T-cell activation.
PD-L1	Programmed cell death 1 ligand 1; Plays a critical role in induction and maintenance of immune tolerance to self. As a ligand for the inhibitory receptor PDCD1/PD-1, modulates the activation threshold of T-cells and limits T-cell effector response. Through a yet unknown activating receptor, may co-stimulate T-cell subsets that predominantly produce interleukin-10 (IL10).
TIGIT	T-cell immunoreceptor with Ig and ITIM domains; Binds with high affinity to the poliovirus receptor (PVR) which causes increased secretion of IL10 and decreased secretion of IL12B and suppresses T-cell activation by promoting the generation of mature immunoregulatory dendritic cells.
CD226	CD226 antigen; Involved in intercellular adhesion, lymphocyte signaling, cytotoxicity and lymphokine secretion mediated by cytotoxic T- lymphocyte (CTL) and NK cell. Cell surface receptor for NECTIN2. Upon ligand binding, stimulates T-cell proliferation and cytokine production, including that of IL2, IL5, IL10, IL13, and IFNG.
TIM-3	Hepatitis A virus cellular receptor 2; Cell surface receptor implicated in modulating innate and adaptive immune responses. Generally accepted to have an inhibiting function. Reports on stimulating functions

	suggest that the activity may be influenced by the cellular context and/or the respective ligand. Regulates macrophage activation. Inhibits T-helper type 1 lymphocyte (Th1)-mediated auto- and allo-immune responses and promotes immunological tolerance.
CD159a	NKG2-A/NKG2-B type II integral membrane protein; Plays a role as a receptor for the recognition of MHC class I HLA-E molecules by NK cells and some cytotoxic T-cells.
bFGF	Fibroblast growth factor 2; Acts as a ligand for FGFR1, FGFR2, FGFR3 and FGFR4. Also acts as an integrin ligand which is required for FGF2 signaling. Binds to integrin ITGAV:ITGB3. Plays an important role in the regulation of cell survival, cell division, cell differentiation and cell migration. Functions as a potent mitogen in vitro. Can induce angiogenesis.
CCL2	C-C motif chemokine 2; Acts as a ligand for C-C chemokine receptor CCR2. Signals through binding and activation of CCR2 and induces a strong chemotactic response and mobilization of intracellular calcium ions. Exhibits a chemotactic activity for monocytes and basophils but not neutrophils or eosinophils. May be involved in the recruitment of monocytes into the arterial wall during the disease process of atherosclerosis.
CCL3	C-C motif chemokine 3; Monokine with inflammatory and chemokinetic properties. Binds to CCR1, CCR4 and CCR5. One of the major HIV-suppressive factors produced by CD8+ T-cells. Recombinant MIP-1-alpha induces a dose- dependent inhibition of different strains of HIV-1, HIV-2, and simian immunodeficiency virus (SIV); Belongs to the intercrine beta (chemokine CC) family.
CCL4	C-C motif chemokine 4; Monokine with inflammatory and chemokinetic properties. Binds to CCR5. One of the major HIV-suppressive factors produced by CD8+ T- cells. Recombinant MIP-1-beta induces a dose-dependent inhibition of different strains of HIV-1, HIV-2, and simian immunodeficiency virus (SIV). The processed form MIP-1-beta(3-69) retains the abilities to induce down-modulation of surface expression of the chemokine receptor CCR5 and to inhibit the CCR5-mediated entry of HIV-1 in T-cells.
CCL5	C-C motif chemokine 5; Chemoattractant for blood monocytes, memory T-helper cells and eosinophils. Causes the release of histamine from basophils and activates eosinophils. May activate several chemokine receptors including CCR1, CCR3, CCR4 and CCR5. One of the major HIV-suppressive factors produced by CD8+ T-cells. Recombinant RANTES protein induces a dose-dependent inhibition of different strains of HIV-1, HIV-2, and simian immunodeficiency virus (SIV). The processed form RANTES(3-68) acts as a natural chemotaxis inhibitor and is a more potent inhibitor of HIV-1-infection.
CCR2	C-C chemokine receptor type 2; Key functional receptor for CCL2 but can also bind CCL7 and CCL12. Its binding with CCL2 on monocytes and macrophages mediates chemotaxis and migration induction through the activation of the PI3K cascade, the small G protein Rac and lamellipodium protrusion (Probable). Also acts as a receptor for the beta-defensin DEFB106A/DEFB106B. Regulates the expression of T-cell inflammatory cytokines and T-cell differentiation, promoting the differentiation of T-cells into T-helper 17 cells (Th17) during inflammation.
CCR5	C-C chemokine receptor type 5; Receptor for a number of inflammatory CC-chemokines including CCL3/MIP-1-alpha, CCL4/MIP-1-beta and RANTES and subsequently transduces a signal by increasing the intracellular calcium ion level. May play a role in the control of granulocytic lineage proliferation or differentiation.
M-CSF	Processed macrophage colony-stimulating factor 1; Cytokine that plays an essential role in the regulation of survival, proliferation and differentiation of hematopoietic precursor cells, especially mononuclear phagocytes, such as macrophages and monocytes. Promotes the release of pro-inflammatory chemokines, and thereby plays an important role in innate immunity and in inflammatory processes. Plays an important role in the regulation of osteoclast proliferation and differentiation, the regulation of bone resorption, and is required for normal bone development.

CXCL9	C-X-C motif chemokine 9; Cytokine that affects the growth, movement, or activation state of cells that participate in immune and inflammatory response. Chemotactic for activated T-cells. Binds to CXCR3; Belongs to the intercrine alpha (chemokine CxC) family.
CXCL10	C-X-C motif chemokine 10; Pro-inflammatory cytokine that is involved in a wide variety of processes such as chemotaxis, differentiation, and activation of peripheral immune cells, regulation of cell growth, apoptosis and modulation of angiostatic effects. Plays thereby an important role during viral infections by stimulating the activation and migration of immune cells to the infected sites.
CXCL11	C-X-C motif chemokine 11; Chemotactic for interleukin-activated T-cells but not unstimulated T-cells, neutrophils or monocytes. Induces calcium release in activated T-cells. Binds to CXCR3. May play an important role in CNS diseases which involve T-cell recruitment.
CXCR3	C-X-C chemokine receptor type 3; [Isoform 1]: Receptor for the C-X-C chemokine CXCL9, CXCL10 and CXCL11 and mediates the proliferation, survival and angiogenic activity of human mesangial cells (HMC) through a heterotrimeric G- protein signaling pathway. Binds to CCL21. Probably promotes cell chemotaxis response. Mediates the activity of CXCL11.
CXCR4	C-X-C chemokine receptor type 4; Receptor for the C-X-C chemokine CXCL12/SDF-1 that transduces a signal by increasing intracellular calcium ion levels and enhancing MAPK1/MAPK3 activation. Involved in the AKT signaling cascade. Plays a role in regulation of cell migration, e.g. during wound healing. Acts as a receptor for extracellular ubiquitin; leading to enhanced intracellular calcium ions and reduced cellular cAMP levels. Binds bacterial lipopolysaccharide (LPS) et mediates LPS-induced inflammatory response, including TNF secretion by monocytes.
EGF	Pro-epidermal growth factor; EGF stimulates the growth of various epidermal and epithelial tissues in vivo and in vitro and of some fibroblasts in cell culture. Magnesiotropic hormone that stimulates magnesium reabsorption in the renal distal convoluted tubule via engagement of EGFR and activation of the magnesium channel TRPM6. Can induce neurite outgrowth in motoneurons of the pond snail Lymnaea stagnalis in vitro.
GDF-15	Growth/differentiation factor 15; Regulates food intake, energy expenditure and body weight in response to metabolic and toxin-induced stresses. Binds to its receptor, GFRAL, and activates GFRAL-expressing neurons localized in the area postrema and nucleus tractus solitarius of the brainstem. It then triggers the activation of neurons localized within the parabrachial nucleus and central amygdala, which constitutes part of the 'emergency circuit' that shapes feeding responses to stressful conditions. On hepatocytes, inhibits growth hormone signaling.
GM-CSF	Granulocyte-macrophage colony-stimulating factor; Cytokine that stimulates the growth and differentiation of hematopoietic precursor cells from various lineages, including granulocytes, macrophages, eosinophils and erythrocytes. Belongs to the GM-CSF family.
IFNA	Interferon alpha-2; Produced by macrophages, IFN-alpha have antiviral activities.
IFNG	Interferon gamma; Produced by lymphocytes activated by specific antigens or mitogens. IFN-gamma, in addition to having antiviral activity, has important immunoregulatory functions. It is a potent activator of macrophages, it has antiproliferative effects on transformed cells and it can potentiate the antiviral and antitumor effects of the type I interferons; Belongs to the type II (or gamma) interferon family.
IL-2	Interleukin-2; Produced by T-cells in response to antigenic or mitogenic stimulation, this protein is required for T-cell proliferation and other activities crucial to regulation of the immune response. Can stimulate B-cells, monocytes, lymphokine-activated killer cells, natural killer cells, and glioma cells.

IL-4	Interleukin-4; Participates in at least several B-cell activation processes as well as of other cell types. It is a co-stimulator of DNA-synthesis. It induces the expression of class II MHC molecules on resting B-cells. It enhances both secretion and cell surface expression of IgE and IgG1. It also regulates the expression of the low affinity Fc receptor for IgE (CD23) on both lymphocytes and monocytes. Positively regulates IL31RA expression in macrophages. Stimulates autophagy in dendritic cells by interfering with mTORC1 signaling and through the induction of RUFY4.
IL-6	Interleukin-6; Cytokine with a wide variety of biological functions. It is a potent inducer of the acute phase response. Plays an essential role in the final differentiation of B-cells into Ig-secreting cells Involved in lymphocyte and monocyte differentiation. Acts on B-cells, T-cells, hepatocytes, hematopoietic progenitor cells and cells of the CNS. Required for the generation of T(H)17 cells. Also acts as a myokine. It is discharged into the bloodstream after muscle contraction and acts to increase the breakdown of fats and to improve insulin resistance.
IL-10	Interleukin-10; Major immune regulatory cytokine that acts on many cells of the immune system where it has profound anti-inflammatory functions, limiting excessive tissue disruption caused by inflammation. Mechanistically, IL10 binds to its heterotetrameric receptor comprising IL10RA and IL10RB leading to JAK1 and STAT2-mediated phosphorylation of STAT3. In turn, STAT3 translocates to the nucleus where it drives expression of anti-inflammatory mediators.
IL-13	Interleukin-13; Cytokine. Inhibits inflammatory cytokine production. Synergizes with IL2 in regulating interferon-gamma synthesis. May be critical in regulating inflammatory and immune responses. Positively regulates IL31RA expression in macrophages (By similarity).
IL-15	Interleukin-15; Cytokine that stimulates the proliferation of T-lymphocytes. Stimulation by IL15 requires interaction of IL15 with components of the IL2 receptor, including IL2RB and probably IL2RG but not IL2RA. In neutrophils, stimulates phagocytosis probably by signaling through the IL15 receptor, composed of the subunits IL15RA, IL2RB and IL2RG, which results in kinase SYK activation ; Belongs to the IL-15/IL-21 family.
IL-21	Interleukin-21; Cytokine with immunoregulatory activity. May promote the transition between innate and adaptive immunity. Induces the production of IgG(1) and IgG(3) in B-cells (By similarity). May play a role in proliferation and maturation of natural killer (NK) cells in synergy with IL15. May regulate proliferation of mature B- and T-cells in response to activating stimuli. In synergy with IL15 and IL18 stimulates interferon gamma production in T-cells and NK cells. During T-cell mediated immune response may inhibit dendritic cells (DC) activation and maturation.
SDF-1	Stromal cell-derived factor 1; Chemoattractant active on T-lymphocytes and monocytes but not neutrophils. Activates the C-X-C chemokine receptor CXCR4 to induce a rapid and transient rise in the level of intracellular calcium ions and chemotaxis. SDF-1-beta(3-72) and SDF-1-alpha(3-67) show a reduced chemotactic activity. Binding to cell surface proteoglycans seems to inhibit formation of SDF-1-alpha(3-67) and thus to preserve activity on local sites.
TGFB	Transforming growth factor beta-1 proprotein; Transforming growth factor beta-1 proprotein: Precursor of the Latency-associated peptide (LAP) and Transforming growth factor beta-1 (TGF-beta-1) chains, which constitute the regulatory and active subunit of TGF-beta-1, respectively. Transforming growth factor beta-1: Multifunctional protein that regulates the growth and differentiation of various cell types and is involved in various processes, such as normal development, immune function, microglia function and responses to neurodegeneration.
TNFA	Tumor necrosis factor, membrane form; Cytokine that binds to TNFRSF1A/TNFR1 and TNFRSF1B/TNFBR. It is mainly secreted by macrophages and can induce cell death of certain tumor cell lines. It is potent pyrogen causing fever by direct action or by stimulation of interleukin-1 secretion and is implicated in the induction of cachexia, Under certain conditions it can stimulate cell proliferation

	and induce cell differentiation. Impairs regulatory T- cells (Treg) function in individuals with rheumatoid arthritis via FOXP3 dephosphorylation.
VEGF	Vascular endothelial growth factor B; Growth factor for endothelial cells. VEGF-B167 binds heparin and neuropilin-1 whereas the binding to neuropilin-1 of VEGF-B186 is regulated by proteolysis.
EGFR	Epidermal growth factor receptor; Receptor tyrosine kinase binding ligands of the EGF family and activating several signaling cascades to convert extracellular cues into appropriate cellular responses. Known ligands include EGF, TGFA/TGF-alpha, AREG, epigen/EPGN, BTC/betacellulin, epiregulin/EREG and HBEGF/heparin- binding EGF. Ligand binding triggers receptor homo- and/or heterodimerization and autophosphorylation on key cytoplasmic residues. The phosphorylated receptor recruits adapter proteins like GRB2 which in turn activates complex downstream signaling cascades.
HER2	Receptor tyrosine-protein kinase erbB-2; Protein tyrosine kinase that is part of several cell surface receptor complexes, but that apparently needs a coreceptor for ligand binding. Essential component of a neuregulin-receptor complex, although neuregulins do not interact with it alone. GP30 is a potential ligand for this receptor. Regulates outgrowth and stabilization of peripheral microtubules (MTs). Upon ERBB2 activation, the MEMO1-RHOA-DIAPH1 signaling pathway elicits the phosphorylation and thus the inhibition of GSK3B at cell membrane.
EphA2	Ephrin type-A receptor 2; Receptor tyrosine kinase which binds promiscuously membrane- bound ephrin-A family ligands residing on adjacent cells, leading to contact-dependent bidirectional signaling into neighboring cells. The signaling pathway downstream of the receptor is referred to as forward signaling while the signaling pathway downstream of the ephrin ligand is referred to as reverse signaling. Activated by the ligand ephrin- A1/EFNA1 regulates migration, integrin-mediated adhesion, proliferation and differentiation of cells.
CD40L	CD40 ligand, membrane form; Cytokine that acts as a ligand to CD40/TNFRSF5. Co-stimulates T-cell proliferation and cytokine production. Its cross-linking on T-cells generates a co-stimulatory signal which enhances the production of IL4 and IL10 in conjunction with the TCR/CD3 ligation and CD28 co-stimulation. Induces the activation of NF-kappa-B. Induces the activation of kinases MAPK8 and PAK2 in T-cells. Induces tyrosine phosphorylation of isoform 3 of CD28. Mediates B-cell proliferation in the absence of co-stimulus as well as IgE production in the presence of IL4.
CD70	CD70 antigen; Cytokine which is the ligand for CD27. The CD70-CD27 pathway plays an important role in the generation and maintenance of T cell immunity, in particular during antiviral responses. Upon CD27 binding, induces the proliferation of co-stimulated T-cells and enhances the generation of cytolytic T-cells.
FasL	Tumor necrosis factor ligand superfamily member 6, membrane form; Cytokine that binds to TNFRSF6/FAS, a receptor that transduces the apoptotic signal into cells. Involved in cytotoxic T-cell-mediated apoptosis, natural killer cell-mediated apoptosis and in T-cell development. Initiates fratricidal/suicidal activation-induced cell death (AICD) in antigen- activated T-cells contributing to the termination of immune responses (By similarity). TNFRSF6/FAS-mediated apoptosis has also a role in the induction of peripheral tolerance.
IL13RA2	Interleukin-13 receptor subunit alpha-2; Binds as a monomer with high affinity to interleukin-13 (IL13), but not to interleukin-4 (IL4).
MHCI	HLA class I histocompatibility antigen, C alpha chain; Antigen-presenting major histocompatibility complex class I (MHCI) molecule with an important role in reproduction and antiviral immunity. In complex with B2M/beta 2 microglobulin displays a restricted repertoire of self and viral peptides and

	acts as a dominant ligand for inhibitory and activating killer immunoglobulin receptors (KIRs) expressed on NK cells.
MHCII	HLA class II histocompatibility antigen, DRB1 beta chain; A beta chain of antigen-presenting major histocompatibility complex class II (MHCII) molecule. In complex with the alpha chain HLA- DRA, displays antigenic peptides on professional antigen presenting cells (APCs) for recognition by alpha-beta T cell receptor (TCR) on HLA-DRB1-restricted CD4-positive T cells. This guides antigen-specific T helper effector functions, both antibody-mediated immune response and macrophage activation, to ultimately eliminate the infectious agents and transformed cells.
NKG2D	NKG2-D type II integral membrane protein; Functions as an activating and costimulatory receptor involved in immunosurveillance upon binding to various cellular stress- inducible ligands displayed at the surface of autologous tumor cells and virus-infected cells. Provides both stimulatory and costimulatory innate immune responses on activated killer (NK) cells, leading to cytotoxic activity. Acts as a costimulatory receptor for T-cell receptor (TCR) in CD8(+) T-cell-mediated adaptive immune responses by amplifying T-cell activation.
STI1	Stress-induced-phosphoprotein 1; Acts as a co-chaperone for HSP90AA1. Mediates the association of the molecular chaperones HSPA8/HSC70 and HSP90.
CD4	T-cell surface glycoprotein CD4; Integral membrane glycoprotein that plays an essential role in the immune response and serves multiple functions in responses against both external and internal offenses. In T-cells, functions primarily as a co-receptor for MHC class II molecule:peptide complex. The antigens presented by class II peptides are derived from extracellular proteins while class I peptides are derived from cytosolic proteins. Interacts simultaneously with the T-cell receptor (TCR) and the MHC class II presented by antigen presenting cells (APCs).
CD8	T-cell surface glycoprotein CD8 alpha chain; Integral membrane glycoprotein that plays an essential role in the immune response and serves multiple functions in responses against both external and internal offenses. In T-cells, functions primarily as a co-receptor for MHC class I molecule:peptide complex. The antigens presented by class I peptides are derived from cytosolic proteins while class II derived from extracellular proteins. Interacts simultaneously with the T-cell receptor (TCR) and the MHC class I proteins presented by antigen presenting cells (APCs).
CD11B	Integrin alpha-M; Integrin ITGAM/ITGB2 is implicated in various adhesive interactions of monocytes, macrophages and granulocytes as well as in mediating the uptake of complement-coated particles and pathogens. It is identical with CR-3, the receptor for the iC3b fragment of the third complement component. It probably recognizes the R-G-D peptide in C3b. Integrin ITGAM/ITGB2 is also a receptor for fibrinogen, factor X and ICAM1. It recognizes P1 and P2 peptides of fibrinogen gamma chain.
CD16	Low affinity immunoglobulin gamma Fc region receptor III-A; Receptor for the Fc region of IgG. Binds complexed or aggregated IgG and also monomeric IgG. Mediates antibody-dependent cellular cytotoxicity (ADCC) and other antibody-dependent responses, such as phagocytosis.
CD25	Interferon-stimulated gene 20 kDa protein; Interferon-induced antiviral exoribonuclease that acts on single-stranded RNA and also has minor activity towards single-stranded DNA. Exhibits antiviral activity against RNA viruses including hepatitis C virus (HCV), hepatitis A virus (HAV) and yellow fever virus (YFV) in an exonuclease-dependent manner. May also play additional roles in the maturation of snRNAs and rRNAs, and in ribosome biogenesis.
CD56	Neural cell adhesion molecule 1; This protein is a cell adhesion molecule involved in neuron- neuron adhesion, neurite fasciculation, outgrowth of neurites, etc.
CD68	Macrosialin; Could play a role in phagocytic activities of tissue macrophages, both in intracellular lysosomal metabolism and extracellular cell-cell and cell-pathogen interactions. Binds to tissue- and

	organ-specific lectins or selectins, allowing homing of macrophage subsets to particular sites. Rapid recirculation of CD68 from endosomes and lysosomes to the plasma membrane may allow macrophages to crawl over selectin-bearing substrates or other cells.
CD133	Prominin-1; May play a role in cell differentiation, proliferation and apoptosis. Binds cholesterol in cholesterol- containing plasma membrane microdomains and may play a role in the organization of the apical plasma membrane in epithelial cells. During early retinal development acts as a key regulator of disk morphogenesis. Involved in regulation of MAPK and Akt signaling pathways. In neuroblastoma cells suppresses cell differentiation such as neurite outgrowth in a RET-dependent manner.
Foxp3	Forkhead box protein P3, C-terminally processed; Transcriptional regulator which is crucial for the development and inhibitory function of regulatory T-cells (Treg). Plays an essential role in maintaining homeostasis of the immune system by allowing the acquisition of full suppressive function and stability of the Treg lineage, and by directly modulating the expansion and function of conventional T-cells. Can act either as a transcriptional repressor or a transcriptional activator depending on its interactions with other transcription factors, histone acetylases and deacetylases.
ERK	Mitogen-activated protein kinase 1; Serine/threonine kinase which acts as an essential component of the MAP kinase signal transduction pathway. MAPK1/ERK2 and MAPK3/ERK1 are the 2 MAPKs which play an important role in the MAPK/ERK cascade. They participate also in a signaling cascade initiated by activated KIT and KITLG/SCF. Depending on the cellular context, the MAPK/ERK cascade mediates diverse biological functions such as cell growth, adhesion, survival and differentiation through the regulation of transcription, translation, cytoskeletal rearrangements.
NFkappaB	Nuclear factor NF-kappa-B p105 subunit; NF-kappa-B is a pleiotropic transcription factor present in almost all cell types and is the endpoint of a series of signal transduction events that are initiated by a vast array of stimuli related to many biological processes such as inflammation, immunity, differentiation, cell growth, tumorigenesis and apoptosis. NF-kappa-B is a homo- or heterodimeric complex formed by the Rel-like domain- containing proteins RELA/p65, RELB, NFKB1/p105, NFKB1/p50, REL and NFKB2/p52 and the heterodimeric p65-p50 complex appears to be most abundant one.
PI3K	Phosphatidylinositol 4,5-bisphosphate 3-kinase catalytic subunit alpha isoform; Phosphoinositide-3-kinase (PI3K) that phosphorylates PtdIns (Phosphatidylinositol), PtdIns4P (Phosphatidylinositol 4-phosphate) and PtdIns(4,5)P2 (Phosphatidylinositol 4,5-bisphosphate) to generate phosphatidylinositol 3,4,5-trisphosphate (PIP3). PIP3 plays a key role by recruiting PH domain-containing proteins to the membrane, including AKT1 and PDPK1, activating signaling cascades involved in cell growth, survival, proliferation, motility and morphology.
AKT	RAC-alpha serine/threonine-protein kinase; AKT1 is one of 3 closely related serine/threonine-protein kinases (AKT1, AKT2 and AKT3) called the AKT kinase, and which regulate many processes including metabolism, proliferation, cell survival, growth and angiogenesis. This is mediated through serine and/or threonine phosphorylation of a range of downstream substrates. Over 100 substrate candidates have been reported so far, but for most of them, no isoform specificity has been reported.
mTOR	Serine/threonine-protein kinase mTOR; Serine/threonine protein kinase which is a central regulator of cellular metabolism, growth and survival in response to hormones, growth factors, nutrients, energy and stress signals. MTOR directly or indirectly regulates the phosphorylation of at least 800 proteins. Functions as part of 2 structurally and functionally distinct signaling complexes mTORC1 and mTORC2 (mTOR complex 1 and 2). Activated mTORC1 up-regulates protein synthesis by phosphorylating key regulators of mRNA translation and ribosome synthesis.
Smad	Mothers against decapentaplegic homolog 1; Transcriptional modulator activated by BMP (bone morphogenetic proteins) type 1 receptor kinase. SMAD1 is a receptor- regulated SMAD (R-SMAD).

	SMAD1/OAZ1/PSMB4 complex mediates the degradation of the CREBBP/EP300 repressor SNIP1. May act synergistically with SMAD4 and YY1 in bone morphogenetic protein (BMP)- mediated cardiac-specific gene expression.
Smad	Mothers against decapentaplegic homolog 2; Receptor-regulated SMAD (R-SMAD) that is an intracellular signal transducer and transcriptional modulator activated by TGF-beta (transforming growth factor) and activin type 1 receptor kinases. Binds the TRE element in the promoter region of many genes that are regulated by TGF-beta and, on formation of the SMAD2/SMAD4 complex, activates transcription. May act as a tumor suppressor in colorectal carcinoma. Positively regulates PDPK1 kinase activity by stimulating its dissociation from the 14-3-3 protein YWHAQ which acts as a negative regulator.
Sonic Hedgehog	Sonic hedgehog protein N-product; [Sonic hedgehog protein]: The C-terminal part of the sonic hedgehog protein precursor displays an autoproteolysis and a cholesterol transferase activity (By similarity). Both activities result in the cleavage of the full-length protein into two parts (ShhN and ShhC) followed by the covalent attachment of a cholesterol moiety to the C-terminal of the newly generated ShhN. Both activities occur in the reticulum endoplasmic. Once cleaved, ShhC is degraded in the endoplasmic reticulum.
MMP-2	72 kDa type IV collagenase; Ubiquitinous metalloproteinase that is involved in diverse functions such as remodeling of the vasculature, angiogenesis, tissue repair, tumor invasion, inflammation, and atherosclerotic plaque rupture. As well as degrading extracellular matrix proteins, can also act on several nonmatrix proteins such as big endothelial 1 and beta- type CGRP promoting vasoconstriction. Also cleaves KISS at a Gly-∣-Leu bond. Appears to have a role in myocardial cell death pathways. Contributes to myocardial oxidative stress by regulating the activity of GSK3beta.
MMP-9	67 kDa matrix metalloproteinase-9; May play an essential role in local proteolysis of the extracellular matrix and in leukocyte migration. Could play a role in bone osteoclastic resorption. Cleaves KiSS1 at a Gly-∣-Leu bond. Cleaves type IV and type V collagen into large C-terminal three quarter fragments and shorter N-terminal one quarter fragments. Degrades fibronectin but not laminin or Pz-peptide. Belongs to the peptidase M10A family.
p53	Cellular tumor antigen p53; Acts as a tumor suppressor in many tumor types; induces growth arrest or apoptosis depending on the physiological circumstances and cell type. Involved in cell cycle regulation as a trans-activator that acts to negatively regulate cell division by controlling a set of genes required for this process. One of the activated genes is an inhibitor of cyclin-dependent kinases. Apoptosis induction seems to be mediated either by stimulation of BAX and FAS antigen expression, or by repression of Bcl-2 expression.
TRAIL	Tumor necrosis factor ligand superfamily member 10; Cytokine that binds to TNFRSF10A/TRAILR1, TNFRSF10B/TRAILR2, TNFRSF10C/TRAILR3, TNFRSF10D/TRAILR4 and possibly also to TNFRSF11B/OPG. Induces apoptosis. Its activity may be modulated by binding to the decoy receptors TNFRSF10C/TRAILR3, TNFRSF10D/TRAILR4 and TNFRSF11B/OPG that cannot induce apoptosis. Belongs to the tumor necrosis factor family.

Table S2. Protein interactions in the glioblastoma microenvironment: functional and physical associations obtained using STRING. According to the String criteria, interactions with a high confidence level (0.700) are displayed.

#node1	#node2	#combined_score
CXCL10	SDF-1	0.999
CXCL10	CXCR3	0.999
CXCL10	CXCL9	0.999
CXCL10	CXCL11	0.999
CXCL10	CXCR4	0.997
CXCL10	TNFA	0.966
CXCL10	IL-10	0.962
CXCL10	IL-6	0.960
CXCL10	IFNG	0.959
CXCL10	GM-CSF	0.911
CXCL10	IL-4	0.882
CXCL10	IL-13	0.871
CXCL10	MMP-9	0.868
CXCL10	IL-2	0.857
CXCL10	IL-15	0.848
CXCL10	NFkappaB	0.845
CXCL10	IFNA	0.838
CXCL10	PD-L1	0.777
CXCL10	PD-1	0.729
CXCL10	M-CSF	0.728
IL-6	NFkappaB	0.994
IL-6	TNFA	0.994
IL-6	TGFB	0.960
IL-6	MMP-9	0.959
IL-6	bFGF	0.882
IL-6	IL13RA2	0.861
IL-6	MMP-2	0.847
IL-6	M-CSF	0.827
IL-6	SDF-1	0.811
IL-6	PD-L1	0.799
IL-6	iNOS	0.781
IL-6	p53	0.777
IL-6	mTOR	0.772
IL-6	TIM-3	0.749
CCL2	CCL5	0.999
CCL2	CCR5	0.999
CCL2	CXCL9	0.999
CCL2	CCR2	0.999
CCL2	CXCR3	0.998
CCL2	CXCR4	0.998
CCL2	IL-6	0.985
CCL2	NFkappaB	0.982
CCL2	TNFA	0.978
CCL2	CXCL10	0.965
CCL2	IL-10	0.963

CCL2	IFNG	0.938
CCL2	IL-13	0.932
CCL2	GM-CSF	0.931
CCL2	IL-4	0.921
CCL2	IL-2	0.853
CCL2	TGFB	0.851
CCL2	SDF-1	0.841
CCL2	CD4	0.839
CCL2	M-CSF	0.833
CCL2	CD68	0.815
CCL2	MMP-9	0.809
CCL2	CD8	0.800
CCL2	CD11B	0.796
CCL2	IL-15	0.796
CCL2	CXCL11	0.796
CCL2	CCL4	0.787
CCL2	FasL	0.737
CCL2	bFGF	0.732
CCL2	CD16	0.725
CCL2	EGFR	0.725
CCL2	IFNA	0.723
CCL2	CD40L	0.714
CCL2	MMP-2	0.704
Arg1	iNOS	0.980
Arg1	IL-10	0.893
Arg1	IL-4	0.880
Arg1	IL-13	0.807
Arg1	IL-6	0.804
Arg1	IFNG	0.801
Arg1	CD11B	0.795
Arg1	TNFA	0.777
Arg1	CD4	0.761
Arg1	CD68	0.742
Arg1	CCR2	0.732
Arg1	CCL2	0.726
Arg1	PD-L1	0.721
Arg1	TIM-3	0.720
Arg1	CD8	0.720
iNOS	Arg1	0.980
iNOS	AKT	0.976
iNOS	IFNG	0.862
iNOS	TNFA	0.858
iNOS	IL-6	0.781
CD11B	CD16	0.948
CD11B	CD4	0.943
CD11B	CD8	0.940
CD11B	IFNG	0.920
CD11B	PI3K	0.916

CD11B	TNFA	0.909
CD11B	IL-6	0.897
CD11B	CD68	0.896
CD11B	CD40L	0.894
CD11B	IL-10	0.856
CD11B	Foxp3	0.855
CD11B	IL-13	0.828
CD11B	GM-CSF	0.811
CD11B	IL-4	0.794
CD11B	CXCR3	0.785
CD11B	M-CSF	0.781
CD11B	CXCL10	0.774
CD11B	PD-L1	0.731
CD11B	CD56	0.727
CD11B	IL-2	0.721
CD11B	FasL	0.715
CD11B	CXCR4	0.715
CD11B	MMP-9	0.713
CD68	CD8	0.893
CD68	TNFA	0.860
CD68	Foxp3	0.850
CD68	IL-6	0.839
CD68	PD-L1	0.823
CD68	IFNG	0.811
CD68	IL-10	0.794
CD68	TGFB	0.787
CD68	MMP-9	0.766
CD68	CXCL10	0.732
CD68	CTLA-4	0.730
CD68	PD-1	0.730
CD68	IL-4	0.724
CD68	M-CSF	0.723
CD68	IL-13	0.645
CD68	GM-CSF	0.633
CD68	HER2	0.612
CD68	TIM-3	0.597
CD68	SDF-1	0.570
CD68	iNOS	0.568
CD68	CXCR4	0.563
CD68	CXCL9	0.554
CD68	IL-2	0.542
CD68	p53	0.542
CD68	NFkappaB	0.541
CD68	LAG-3	0.503
CD68	bFGF	0.503
CD68	MMP-2	0.495
CD68	IL-15	0.462
CD68	CXCR3	0.461

CD68	CXCL11	0.455
CD68	EGF	0.449
CD68	EGFR	0.449
CD68	FasL	0.411
CCR2	SDF-1	0.998
CCR2	CXCL10	0.998
CCR2	CCR5	0.993
CCR2	CXCL9	0.993
CCR2	CXCR4	0.989
CCR2	CXCR3	0.970
CCR2	CXCL11	0.948
CCR2	CD4	0.909
CCR2	IL-6	0.879
CCR2	IL-10	0.878
CCR2	CD8	0.840
CCR2	IFNG	0.837
CCR2	M-CSF	0.837
CCR2	CD11B	0.826
CCR2	CD68	0.788
CCR2	TNFA	0.771
CCR2	CD16	0.761
CCR2	IL-2	0.751
CCR2	CTLA-4	0.725
MMP-2	TGFB	0.962
MMP-2	MMP-9	0.918
MMP-2	bFGF	0.802
MMP-2	SDF-1	0.762
MMP-2	TNFA	0.729
MMP-9	TGFB	0.968
MMP-9	TNFA	0.930
MMP-9	bFGF	0.919
MMP-9	SDF-1	0.891
MMP-9	p53	0.838
MMP-9	NFkappaB	0.802
IL-4	IL13RA2	0.992
IL-4	IL-6	0.986
IL-4	TNFA	0.977
IL-4	TGFB	0.852
IL-4	NF-kappa B	0.800
IL-4	M-CSF	0.791
IL-4	SDF-1	0.771
IL-4	mTOR	0.744
IL-4	TIM-3	0.719
IL-4	PD-L1	0.718
IL-4	p53	0.718
TGFB	bFGF	0.982
TGFB	TNFA	0.941
TGFB	p53	0.809

M-CSF	TNFA	0.801
M-CSF	TGFB	0.776
M-CSF	SDF-1	0.701
CD4	CD8	0.999
CD4	CXCR4	0.999
CD4	MHCII	0.994
CD4	IFNG	0.991
CD4	CD40L	0.983
CD4	Foxp3	0.979
CD4	IL-10	0.976
CD4	IL-2	0.975
CD4	IL-4	0.970
CD4	TNFA	0.968
CD4	IL-6	0.966
CD4	PD-1	0.960
CD4	CTLA-4	0.955
CD4	PD-L1	0.938
CD4	IL-13	0.931
CD4	IL-15	0.917
CD4	LAG-3	0.914
CD4	CD68	0.898
CD4	CD56	0.883
CD4	GM-CSF	0.880
CD4	TGFB	0.870
CD4	FasL	0.868
CD4	CXCR3	0.864
CD4	NF-kappa B	0.858
CD4	MHCI	0.857
CD4	CXCL10	0.839
CD4	TIM-3	0.833
CD4	SDF-1	0.825
CD4	CXCL9	0.790
CD4	IFNA	0.788
CD4	NKG2D	0.780
CD4	CXCL11	0.769
CD4	Tigit	0.757
CD4	IL-21	0.716
CD4	p53	0.703
CD4	MMP-9	0.701
CD8	MHCI	0.994
CD8	IFNG	0.990
CD8	IL-2	0.971
CD8	IL-10	0.958
CD8	TNFA	0.958
CD8	Foxp3	0.948
CD8	LAG-3	0.947
CD8	IL-4	0.947
CD8	NKG2D	0.946

CD8	CTLA-4	0.940
CD8	PD-L1	0.939
CD8	PD-1	0.938
CD8	IL-15	0.936
CD8	IL-6	0.930
CD8	FasL	0.922
CD8	CXCR3	0.910
CD8	CXCR4	0.903
CD8	TIM-3	0.892
CD8	CXCL10	0.887
CD8	GM-CSF	0.873
CD8	Tigit	0.861
CD8	IL-13	0.854
CD8	CXCL9	0.848
CD8	NF-kappa B	0.818
CD8	CXCL11	0.800
CD8	TGFB	0.796
CD8	MHCII	0.785
CD8	p53	0.770
CD8	IFNA	0.759
CD8	HER2	0.720
CD8	SDF-1	0.701
CD159a	MHCI	0.988
CD159a	PD-L1	0.888
CD159a	CD226	0.873
CD159a	NKG2D	0.847
CD159a	CD4	0.839
CD159a	Tigit	0.826
CD159a	CD56	0.803
CD159a	CD16	0.796
CD159a	IFNG	0.786
CD159a	CD8	0.774
CD159a	PD-1	0.743
CD159a	IL-15	0.733
CD159a	CTLA-4	0.728
CD159a	TIM-3	0.711
CD159a	IL-2	0.710
CD226	Tigit	0.986
CD226	NKG2D	0.936
CD226	CD56	0.701
Foxp3	IL-2	0.991
Foxp3	IFNG	0.982
Foxp3	IL-10	0.955
Foxp3	IL-4	0.925
Foxp3	IL-6	0.913
Foxp3	PD-L1	0.912
Foxp3	TNFA	0.909
Foxp3	TGFB	0.865

Foxp3	Tigit	0.822
Foxp3	IL-13	0.808
Foxp3	LAG-3	0.807
Foxp3	IL-15	0.807
Foxp3	PD-1	0.799
Foxp3	TIM-3	0.787
Foxp3	NF-kappa B	0.744
Foxp3	GM-CSF	0.743
IL-2	IL-4	0.999
IL-2	IL-6	0.999
IL-2	TNFA	0.957
IL-2	PD-L1	0.854
IL-2	PD-1	0.850
IL-2	NKG2D	0.847
IL-2	NFkappaB	0.804
IL-2	IL13RA2	0.792
IL-2	TGFB	0.763
IL-2	LAG-3	0.744
IL-2	p53	0.741
IL-2	bFGF	0.740
IL-2	TIM-3	0.737
IL-2	IL-21	0.734
IL-21	IL-4	0.719
CD16	CD56	0.991
CD16	CD4	0.964
CD16	NKG2D	0.954
CD16	CD8	0.949
CD16	IFNG	0.926
CD16	IL-15	0.924
CD16	IL-2	0.878
CD16	PD-L1	0.878
CD16	TNFA	0.872
CD16	HER2	0.839
CD16	PD-1	0.833
CD16	Foxp3	0.822
CD16	IL-10	0.805
CD16	IL-6	0.796
CD16	IL-4	0.787
CD16	EGFR	0.778
CD16	CD68	0.774
CD16	GM-CSF	0.769
CD16	CTLA-4	0.767
CD16	CXCR4	0.757
CD16	CD226	0.726
CD16	CXCL10	0.711
CD16	TIM-3	0.703
CD56	CD8	0.889
CD56	IFNG	0.863

CD56	IL-2	0.856
CD56	IL-15	0.841
CD56	NKG2D	0.830
CD56	CD68	0.721
CD56	Foxp3	0.708
CD56	IL-10	0.702
CD56	TNFA	0.701
IFNA	IFNG	0.968
IFNA	IL-6	0.903
IFNA	IL-2	0.892
IFNA	IL-10	0.890
IFNA	NF-kappa B	0.863
IFNA	bFGF	0.863
IFNA	TNFA	0.842
IFNA	IL-15	0.779
IFNG	IL-10	0.995
IFNG	TNFA	0.991
IFNG	IL-2	0.990
IFNG	IL-4	0.990
IFNG	IL-6	0.983
IFNG	IL-13	0.961
IFNG	IL-15	0.939
IFNG	NF-kappa B	0.932
IFNG	PD-1	0.915
IFNG	PD-L1	0.899
IFNG	TGFB	0.888
IFNG	TIM-3	0.885
IFNG	LAG-3	0.877
IFNG	IL13RA2	0.876
IFNG	iNOS	0.862
IFNG	NKG2D	0.841
IFNG	p53	0.834
IFNG	MHCII	0.830
IFNG	M-CSF	0.790
IFNG	Tigit	0.767
IFNG	MMP-9	0.751
IFNG	IL-21	0.747
IFNG	SDF-1	0.713
GM-CSF	IL-2	0.997
GM-CSF	IFNG	0.984
GM-CSF	IL-6	0.974
GM-CSF	TNFA	0.970
GM-CSF	bFGF	0.965
GM-CSF	M-CSF	0.964
GM-CSF	IL-4	0.963
GM-CSF	IL-10	0.959
GM-CSF	IL-13	0.934
GM-CSF	IFNA	0.868

GM-CSF	IL-15	0.849
GM-CSF	IL13RA2	0.771
GM-CSF	PD-L1	0.748
GM-CSF	NKG2D	0.740
GM-CSF	NF-kappa B	0.730
GM-CSF	p53	0.718
GM-CSF	TGFB	0.701
CXCR4	SDF-1	0.999
CXCR4	IL-6	0.921
CXCR4	MMP-9	0.844
CXCR4	EGF	0.825
CXCR4	TNFA	0.814
CXCR4	bFGF	0.795
CXCR4	TGFB	0.794
CXCR4	M-CSF	0.766
CXCR4	PD-L1	0.752
CXCR4	NF-kappa B	0.734
CXCR4	Foxp3	0.731
CXCR4	HER2	0.729
CXCR4	IFNG	0.711
IL-10	IL-6	0.997
IL-10	TNFA	0.992
IL-10	IL-4	0.986
IL-10	IL-2	0.964
IL-10	IL-13	0.952
IL-10	TGFB	0.932
IL-10	IL-15	0.914
IL-10	MMP-9	0.881
IL-10	NF-kappa B	0.877
IL-10	PD-L1	0.877
IL-10	M-CSF	0.870
IL-10	TIM-3	0.862
IL-10	PD-1	0.827
IL-10	bFGF	0.822
IL-10	IL13RA2	0.793
IL-10	LAG-3	0.763
IL-10	MMP-2	0.761
IL-10	p53	0.756
IL-10	mTOR	0.740
IL-10	Tigit	0.740
IL-10	SDF-1	0.725
IL-10	iNOS	0.718
IL-13	IL13RA2	0.999
IL-13	IL-4	0.993
IL-13	TNFA	0.957
IL-13	IL-6	0.957
IL-13	IL-2	0.934
IL-13	IL-15	0.871

IL-13	TGFB	0.816
IL-13	MMP-9	0.759
IL-13	M-CSF	0.733
IL-13	bFGF	0.717
IL-15	IL-2	0.958
IL-15	NKG2D	0.921
IL-15	IL-4	0.915
IL-15	TNFA	0.885
IL-15	IL-6	0.884
IL-15	IL13RA2	0.781
IL-15	PD-L1	0.717
IL-15	IL-21	0.714
TNFA	TRAIL	0.946
TNFA	p53	0.865
TNFA	iNOS	0.858
TNFA	bFGF	0.732
CD133	EGFR	0.801
CD133	HER2	0.770
CD133	CXCR4	0.728
CD133	EGF	0.725
CD133	bFGF	0.720
CXCL11	SDF-1	0.999
CXCL11	CXCL9	0.999
CXCL11	CXCR3	0.999
CXCL11	CXCR4	0.994
CXCL11	TGFB	0.947
CXCL11	IFNG	0.835
CXCL11	IL-6	0.735
CXCL11	IL-10	0.724
CXCL11	TNFA	0.705
CXCL9	CXCR3	0.999
CXCL9	SDF-1	0.999
CXCL9	CXCR4	0.994
CXCL9	IFNG	0.948
CXCL9	IL-10	0.819
CXCL9	TNFA	0.815
CXCL9	IL-6	0.815
CXCL9	IFNA	0.802
CXCL9	IL-15	0.771
CXCL9	PD-L1	0.761
CXCL9	IL-2	0.738
CXCL9	GM-CSF	0.736
CXCL9	IL-4	0.735
CXCL9	IL-13	0.722
CXCR3	SDF-1	0.998
CXCR3	CXCR4	0.969
CXCR3	IFNG	0.914
CXCR3	Foxp3	0.846

CXCR3	TNFA	0.817
CXCR3	IL-10	0.804
CXCR3	LAG-3	0.803
CXCR3	IL-4	0.785
CXCR3	Tigit	0.785
CXCR3	IL-6	0.759
CXCR3	IL-2	0.721
CXCR3	PD-1	0.715
CCL3	CCR2	0.999
CCL3	CCR5	0.999
CCL3	CXCR3	0.997
CCL3	CCL4	0.997
CCL3	CXCR4	0.993
CCL3	TNFA	0.988
CCL3	IL-10	0.972
CCL3	IL-6	0.962
CCL3	CXCL10	0.951
CCL3	IFNG	0.947
CCL3	GM-CSF	0.935
CCL3	CCL5	0.913
CCL3	IL-13	0.895
CCL3	CXCL9	0.884
CCL3	CD4	0.880
CCL3	IL-2	0.876
CCL3	CD8	0.873
CCL3	CXCL11	0.851
CCL3	IL-4	0.827
CCL3	SDF-1	0.823
CCL3	IL-15	0.809
CCL3	CD40L	0.799
CCL3	NF-kappa B	0.749
CCL3	M-CSF	0.728
CCL3	CD11B	0.711
CCL4	CCR2	0.999
CCL4	CCR5	0.999
CCL4	CXCR3	0.995
CCL4	CXCR4	0.995
CCL4	TNFA	0.967
CCL4	CXCL10	0.959
CCL4	CXCL9	0.959
CCL4	CCL5	0.953
CCL4	SDF-1	0.948
CCL4	IFNG	0.942
CCL4	CD4	0.939
CCL4	IL-10	0.936
CCL4	IL-6	0.933
CCL4	GM-CSF	0.924
CCL4	CXCL11	0.917

CCL4	CD8	0.889
CCL4	CD40L	0.852
CCL4	IL-13	0.830
CCL4	IL-2	0.824
CCL4	IL-15	0.808
CCL4	IL-4	0.805
CCL4	LAG-3	0.735
CCL5	CCR2	0.999
CCL5	CCR5	0.999
CCL5	CXCL10	0.999
CCL5	CXCL11	0.999
CCL5	CXCL9	0.999
CCL5	CXCR3	0.999
CCL5	SDF-1	0.999
CCL5	CXCR4	0.998
CCL5	NF-kappa B	0.975
CCL5	TNFA	0.960
CCL5	CD8	0.959
CCL5	IL-6	0.950
CCL5	IL-10	0.945
CCL5	GM-CSF	0.922
CCL5	IFNG	0.921
CCL5	CD4	0.887
CCL5	IL-13	0.834
CCL5	CD11B	0.833
CCL5	IL-4	0.828
CCL5	IL-2	0.813
CCL5	MMP-9	0.813
CCL5	IL-15	0.812
CCL5	FasL	0.790
CCL5	M-CSF	0.732
CCL5	CD40L	0.731
CCL5	EGF	0.719
CCL5	CD16	0.703
CCR5	CD4	0.999
CCR5	CXCL10	0.999
CCR5	SDF-1	0.998
CCR5	CXCL9	0.998
CCR5	CXCR4	0.996
CCR5	CXCL11	0.990
CCR5	CXCR3	0.983
CCR5	CD8	0.941
CCR5	IFNG	0.920
CCR5	TNFA	0.880
CCR5	IL-10	0.878
CCR5	CD11B	0.840
CCR5	IL-6	0.831
CCR5	CD68	0.806

CCR5	CD16	0.792
CCR5	IL-4	0.781
CCR5	CD40L	0.771
CCR5	IL-2	0.758
CCR5	IFNA	0.757
CCR5	GM-CSF	0.754
CCR5	LAG-3	0.736
CCR5	Foxp3	0.701
PD-1	PD-L1	0.999
PD-L1	TIM-3	0.998
PD-L1	Tigit	0.973
PD-1	TIM-3	0.891
PD-1	Tigit	0.846
PD-L1	TNFA	0.807
PD-L1	TGFB	0.781
PD-L1	p53	0.776
PD-L1	SDF-1	0.757
PD-L1	mTOR	0.711
PD-1	TNFA	0.700
CTLA-4	PD-L1	0.999
CTLA-4	PD-1	0.992
CTLA-4	Foxp3	0.976
CTLA-4	LAG-3	0.965
CTLA-4	IFNG	0.951
CTLA-4	IL-2	0.943
CTLA-4	IL-10	0.922
CTLA-4	Tigit	0.922
CTLA-4	TIM-3	0.907
CTLA-4	IL-4	0.875
CTLA-4	TNFA	0.873
CTLA-4	FasL	0.833
CTLA-4	IL-6	0.815
CTLA-4	GM-CSF	0.750
CTLA-4	MHCII	0.746
CTLA-4	CXCR3	0.737
CTLA-4	CXCL9	0.703
CD70	TNFA	0.747
CD70	CTLA-4	0.744
CD70	TIM-3	0.741
B7-H3	CTLA-4	0.985
B7-H3	CD40L	0.935
B7-H3	CD4	0.877
B7-H3	CD8	0.873
B7-H3	IL-2	0.828
B7-H3	IL-4	0.814
B7-H3	CD70	0.797
B7-H3	PD-1	0.784
B7-H3	IFNG	0.780

B7-H3	TNFA	0.745
B7-H3	TIM-3	0.736
B7-H3	LAG-3	0.734
B7-H3	IL-10	0.734
B7-H3	Foxp3	0.729
LAG-3	PD-L1	0.976
LAG-3	Tigit	0.955
LAG-3	PD-1	0.950
LAG-3	TIM-3	0.922
LAG-3	MHCII	0.750
SDF-1	bFGF	0.896
SDF-1	TGFB	0.798
SDF-1	TNFA	0.750
TIM-3	Tigit	0.897
TIM-3	TNFA	0.779
bFGF	p53	0.754
EGF	HER2	0.999
EGF	EGFR	0.999
EGF	bFGF	0.969
EGF	M-CSF	0.942
EGF	TGFB	0.904
EGF	GM-CSF	0.891
EGF	IL-6	0.890
EGF	IL-2	0.814
EGF	EphA2	0.802
EGF	TNFA	0.793
EGF	SDF-1	0.790
EGF	ERK	0.789
EGF	Sonic Hedgehog	0.783
EGF	IFNG	0.781
EGF	p53	0.780
EGF	PI3K	0.746
EGF	MMP-9	0.741
EGF	IL13RA2	0.739
EGF	IL-10	0.701
EGFR	PI3K	0.999
EGFR	HER2	0.999
EGFR	IL-6	0.979
EGFR	TGFB	0.975
EGFR	bFGF	0.957
EGFR	p53	0.943
EGFR	M-CSF	0.941
EGFR	EphA2	0.903
EGFR	IL-10	0.899
EGFR	SDF-1	0.897
EGFR	TNFA	0.890
EGFR	IFNG	0.876
EGFR	PD-L1	0.870

EGFR	IL-2	0.851
EGFR	GM-CSF	0.842
EGFR	NF-kappa B	0.837
EGFR	MMP-9	0.827
EGFR	IL-4	0.820
EGFR	IL-13	0.809
EGFR	VEGF	0.805
EGFR	mTOR	0.802
EGFR	IL-15	0.793
EGFR	IFNA	0.778
EGFR	Sonic Hedgehog	0.777
EGFR	IL13RA2	0.775
EGFR	Smad	0.736
EGFR	PD-1	0.705
EphA2	HER2	0.834
EphA2	IL13RA2	0.801
EphA2	bFGF	0.773
EphA2	M-CSF	0.738
EphA2	VEGF	0.736
GDF-15	p53	0.762
HER2	PI3K	0.992
HER2	p53	0.935
HER2	bFGF	0.857
HER2	IL13RA2	0.851
HER2	PD-L1	0.798
HER2	M-CSF	0.792
HER2	NKG2D	0.782
HER2	VEGF	0.777
HER2	IL-6	0.752
HER2	MMP-2	0.738
HER2	NF-kappa B	0.733
HER2	mTOR	0.725
MHCI	MHCII	0.967
MHCI	TNFA	0.794
MHCII	TNFA	0.842
MHCII	PD-1	0.769
NKG2D	PD-1	0.739
NKG2D	Tigit	0.711
